# Validation of visual analogue scales to assess occupational stress compared to the Karasek questionnaire: A cross sectional study

**DOI:** 10.1371/journal.pone.0340209

**Published:** 2026-02-10

**Authors:** Maëlys Clinchamps, Bruno Pereira, Martial Mermillod, Morteza Charkhabi, Marek Zak, Jiao Jiao, Alistair Cole, Jean-Baptiste Bouillon-Minois, Frédéric Dutheil

**Affiliations:** 1 Université Clermont Auvergne, CNRS, LaPSCo, Physiological and Psychosocial Stress, University Hospital of Clermont–Ferrand, CHU Clermont–Ferrand, Occupational and Environmental Medicine, WittyFit, Clermont–Ferrand, France; 2 University Hospital of Clermont Ferrand, CHU Clermont–Ferrand, Clinical Research and Innovation Direction, Clermont-Ferrand, France; 3 Univ. Grenoble Alpes, Univ. Savoie Mont Blanc, Laboratoire de Psychologie et NeuroCognition (LPNC), CNRS, Grenoble, France; 4 HSE University, Moscow, Russia; 5 The Jan Kochanowski University, Faculty of Medicine and Health Sciences, Institute of Physiotherapy, Kielce, Poland; 6 Department of Sports and Health Sciences, Academy of Wellness and Human Development, Hong Kong Baptist University, Sport and Physical Education, Hong Kong, China; 7 Sciences Po Lyon, laboratoire TRIANGLE, Lyon, France; 8 University Hospital of Clermont-Ferrand, Emergency Medicine, Clermont-Ferrand, France; STIKES Wira Medika PPNI Bali: Sekolah Tinggi Ilmu Kesehatan Wira Medika PPNI Bali, INDONESIA

## Abstract

**Background:**

The Job Demand-Control-Support (JDCS) model is one of the most important tools for assessing work-related stress. However, its complexity highlights the need for simpler instruments, such as the Visual Analog Scale (VAS), for rapid assessment in occupational medicine.

**Objectives:**

To validate three VAS corresponding to the main JDCS dimensions: job demand, job control, and social support.

**Method:**

We conducted an observational cross-sectional validation study using a self-administered questionnaire completed twice, a week apart, at the participants’ convenience, to perform test-retest.

**Results:**

We analysed 155 participants (60 for test and retest), mostly women around 40 years. Acceptability was excellent, with high response rates. Internal consistency analysis revealed moderate correlations between VAS and JDCS model main dimensions. Reliability assessed by Lin’s concordance correlation coefficient was acceptable for the VAS and higher for the JDCS. Mean VAS scores indicated significant differences between low and high demand, control, and social support, with cut-off values of 58, 71.5 and 63.5 respectively. For external validity, we mainly found high agreement between VAS and JDCS.

**Conclusions:**

VAS are valid, quick, easy to use, and reliable tools for the assessment of job demand, job control and social support in daily clinical practice for primary prevention and diagnosis. Based on our findings, easier-to-remember cut-offs could be proposed at 60, 70, and 60 for VAS job demand, VAS job control, and VAS social support, respectively. However, when results are over the determined cut-off, we encourage the use of JDCS questionnaire.

**Trial registration:**

ClinicalTrials.gov NCT05871411.

## Introduction

The constant changes in our societies have led to changes in the organization and working conditions [1]. These rapid transformations, to meet economic challenges, improve efficacy and efficiency, have favoured the appearance of work environments that are sometimes poorly organized and managed [2]. It is well established in the literature that stressors in the workplace can consume psychological resources and lead to the appearance of psychosocial risks [3] or work-related stress [4]. Psychosocial risks emerge from the interplay between an individual’s psychological factors and the social aspects of their work environment. These risks can lead to various outcomes, among which is the development of chronic stress syndrome. Work-related stress increases the likelihood of chronic illnesses, including cardiovascular issues and mental health disorders [5,6]. Given that adults typically spend half of their waking hours at work, the workplace serves as a crucial environment for fostering health and well-being [7]. In recent decades, the interest for researchers for occupational stress has grown, leading to the development and validation of various questionnaires, scales, and assessment tools. Nowadays, one of the main models for assessing stress in the workplace is the Job Demand-Control-Support (JDCS) formulated and validated by Karasek [3,8]. This model focuses on the significant impact of daily environmental stressors on long-term stress experiences [9]. Initially, the model was two-dimensional, taking into account psychological demand and job control. The combination of high psychological demand (high workload, time constraints, etc.) and low decision-making latitude (little autonomy and use of skills) creates a situation of high psychological tension at work, known as “job strain”. Job strain is considered a risk factor for workers’ physical and mental health. In 1982, Karasek added social support as a third dimension, recognizing the importance of social relations at work in stress management. Indeed, social support at work (support from colleagues and superiors) can mitigate the harmful effects of job strain on health. Low social support combined with high job strain increases the risk and puts people in a situation known as isostrain [10,11]. The Job Content Questionnaire (JCQ), based on Karasek’s model, has been developed and validated in multiple languages [12,13]. Although it is widely used in the research field, this tool is challenging to use in occupational medicine consultations. Indeed, due to its length (26 items) and complexity, attention and concentration are reduced, leading to a drop in the response rate correlated with the length of the questionnaire. Self-reported questionnaires presented several limits, ranging from low level of completion and participation [14,15], low level of representativeness [16] or high level of missing data [17]. Occupational physicians face time constraints due to the large number of workers and worksites they oversee [14]. Considering that work-related stress is a significant public health issue, it is important for occupational health services to act as gateways for diagnosing psychological risk at work [15]. Rapid, simple screening tools are essential for preventive occupational health. Several brief instruments have been developed to facilitate the assessment of occupational stress, such as short forms of validated questionnaires like the 10-item Perceived Stress Scale (PSS-10) [18], the Single-Item Stress Question (SISQ) [19], or the Stress Visual Analogue Scale [20,21]. While these instruments provide quick estimates of general stress levels, few have been explicitly designed to reflect the theoretical structure of the JDCS model, which remains the cornerstone of occupational stress research. Existing studies using VAS for work-related stress mainly focus on global stress perception or job satisfaction, rather than specific JDCS dimensions such as demand, control, or social support. A visual analogue scale (VAS) is a tool consisting of a continuous line, typically 100 mm long, anchored by two opposite descriptors (e.g., “very low” to “very high”), on which respondents indicate the intensity of their perceived experience. Although they have some limitations (subjective interpretation, lack of detail, etc.), visual analogue scales (VAS) are well-recognized and validated tool with satisfying psychometric properties. They are simple to use, quick, reproducible, sensitive to variations and offer a wide choice of responses that cannot be memorized by the patient between assessment [20–23]. The present study aims to address this gap by validating three VAS corresponding to the JDCS dimensions, offering a theoretically grounded yet time-efficient alternative for occupational health practice. Hence, we hypothesized that using VAS for JDCS dimensions (demand, control, support, job strain) would provide suitable instruments for identifying workers vulnerable to work-related stress, compared with the Karasek questionnaire. Moreover, the JDCS explores social support through two subdimensions: support from colleagues and support from hierarchy, i.e., direct supervisor. Previous research in organizational psychology has shown that perceived organizational support is strongly associated with reduced stress, greater job satisfaction, and improved mental health outcomes [24,25]. Integrating this “company support” dimension within the JDCS framework broadens the model to include both interpersonal and institutional sources of support, enhancing its relevance in large organizations where management culture strongly shapes stress experiences.

The primary objective was to validate three VAS for the main dimensions of the JDCS model (job demand, job control, social support). The secondary objective was to validate three VAS for the subdimensions of social support: support from colleagues (colleague support), support from direct supervisor (head support), and support from the company (company support).

## Methods

### Study design

We conducted an observational cross-sectional validation study by distributing a self-administered questionnaire to volunteers via the REDCap (Research Electronic Data Capture) software platform. REDCap is a secure, web-based software platform designed to support data capture for research studies, providing 1) an intuitive interface for validated data capture; 2) audit trails for tracking data manipulation and export procedures; 3) automated export procedures for seamless data downloads to common statistical packages; and 4) procedures for data integration and interoperability with external sources [26,27]. The REDCap questionnaire was hosted by the University Hospital of Clermont-Ferrand. All participants were informed of the objective of the survey and were volunteers to participate. This exploratory study was undertaken in an ecological setting and received approval from the National Commission for Information Technology and Civil Liberties (CNIL) and from the Ethics Committee Est IV, Strasbourg, France (clinicaltrials.gov identifier NCT05871411). Data were collected anonymously, no personal or identifying information was stored, and participants were informed that they could withdraw at any time without any consequence. The ethics committee waived the requirement for written consent, considering that participants provide their consent by completing the questionnaires on the website.

### Participants

The participants were workers from all sectors, recruited in France. The only inclusion criterion was having a professional activity and there were no specific exclusion criteria. Participants were included between June 13, 2023 and September 13, 2023.

### Main outcomes

We used six VAS and the Job Content Questionnaire (JCQ) of Karasek derived from the JDCS model as the gold standard [3]. The six **VAS** were “Job demand”, “Job control”, “Social support at work”, “Head support”, “Company support” and “Colleagues support”. VAS measured individuals’ feelings at work on a horizontal, uncalibrated 100 mm line, ranging from very low (0) to very high (100). The JCQ is a 26-item questionnaire assessing “Job demand”, “Job control”, “Hierarchy support”, “Colleagues support”. Score for each dimension were calculated according to standard procedures. Job strain and isostrain were defined based on established thresholds derived from French data. The complete definitions and scoring for both JDCS and VAS are provided in the S1 File.

### Outcomes for external validity

Secondary outcomes were sociodemographic (age, sex, height, body mass index – BMI, marital status, number of children, level of education), characteristics of work (occupation, working hours, management function), lifestyle behavior (smoking, alcohol) and mental health (levels of anxiety and depression assess using the hospital and anxiety scale (HADS) [28].

### Time of measurements

The participants completed the questionnaires twice, one week apart, at the most convenient time of day for them, to perform test-retest. The one-week interval was chosen in accordance with methodological recommendations stating that the interval between repeated administrations should be long enough to prevent recall bias but short enough to avoid genuine clinical or contextual changes. A one- to two-week interval is generally considered appropriate, provided that it is clearly described and justified [29,30]. The total completion time was around 30 minutes, with the first session lasting about 20 minutes and the second session about 10 minutes.

### Statistics

Sample size was determined according to COSMIN recommendations: 1) “Rules-of-thumb vary from four to ten subjects per variable, with a minimum number of 100 workers to ensure stability of the variance-covariance matrix” and 2) “Often 0.70 is recommended as a minimum standard for reliability [30,31]. We gave a positive rating for reliability when the intraclass correlation coefficient (ICC) or weighted Kappa was at least 0.70 in a sample size of at least 50 patients.” **Participant’s characteristics** were expressed as means ± standard-deviation (SD) or median [interquartile range] for continuous data (assumption of normality assessed using the Shapiro-Wilk test) and as number (%) for categorical parameters. The analyses were conducted according to COSMIN recommendations following the usual steps of validation of a new questionnaire [31]. The **acceptability** of VAS was assessed with detailed descriptive analysis including the statistical distribution: mean, standard-deviation, median, interquartile range, skewness, kurtosis. The **internal consistency** (interrelatedness among VAS) and the **content validity** (relationship between VAS and gold-standard, i.e., dimensions of the JDCS model as gold-standard: job demand, job control, social support) were evaluated using correlation coefficients (Pearson or Spearman, according to statistical distribution) and principal component analysis (PCA). The dimensions of the JDCS model were first treated as continuous variables. Then, the dimensions were categorized according to threshold reported in literature. The relationships between VAS and dimensions of the JDCS model were analysed using Student t-test or Mann-Whitney test when the assumptions of t-test were not met. The equality of variances was analysed using Fisher-Snedecor test. A specific attention was given to the magnitude of differences. The results were expressed using Hedges’ effect-size (ES) with 95% confidence interval (95 CI) and were interpreted according to the recommendations of Cohen, who defined the ES bounds as small (ES = 0.2), medium (ES = 0.5), and large (ES = 0.8). Furthermore, a receiver operating characteristic (ROC) curve analysis was proposed to determine the best thresholds of VAS to predict a gold-standard JDCS from Karasek, according to clinical relevance and usual indexes reported in the literature (Youden, Lin and efficiency). Sensitivity, specificity, positive and negative predictive values (PPV and NPV) were calculated and presented with 95 CI. The agreement between VAS and dimensions of the JDCS model, treated as categorical variables, was analysed using percentage of agreement and Cohen kappa coefficient. The concordance between JDCS quadrants of Karasek and their equivalents from VAS, according to cut-offs determined by ROC curve analysis, was evaluated using agreement rate and Kappa concordance coefficient. **Test-retest reproducibility** was assessed using Lin concordance correlation coefficient and Bland and Altman plots [32] for VAS as continuous variables. In addition, we performed a sensitivity analysis comparing test–retest reliability coefficients between participants who completed the retest within 7 days (n = 28) and those who completed it after 7 days (n = 27), in order to assess the potential influence of the test–retest interval on stability of the results. Percentage of agreement and Cohen kappa coefficient were used for VAS as categorical variables defined according to aforementioned analyses. **External validity** was assessed by taking into account the generalizability of the new scales, i.e., relation with other variables or groups. More specifically, external validity for VAS as continuous variables were assessed using correlation with secondary outcomes (such as relation between VAS and sociodemographic or psychological measures), and ANOVA or Kruskal-Wallis test if ANOVA assumptions were not met. When appropriate, post-hoc tests were performed considering multiple comparisons (Tukey-Kramer post ANOVA and Dunn after Kruskal-Wallis). External validity for VAS as qualitative variables (prevalence below and above cut-offs determined by ROC curve for each dimension), as well as prevalence of jobstrain and isostrain, were carried out using the Chi-squared or Fisher’s exact test. When more than two groups, a post-hoc test was used (Marascuilo procedure). In order to paid specific attention on the magnitude of differences and to the clinical relevance, the results were expressed as using Cramer’s V, regression coefficient (from linear regression when using outcomes as quantitative variables: job demand, job control, social support) and odds ratio (from logistic regression when using outcomes as qualitative variable: jobstrain, isostrain –) in addition to p-values. All analyses were performed using Stata software (Version 15, StataCorp, College Station, TX) for a two-sided Type I error of α = 5%.

## Results

### Participants

**Fig 1:**
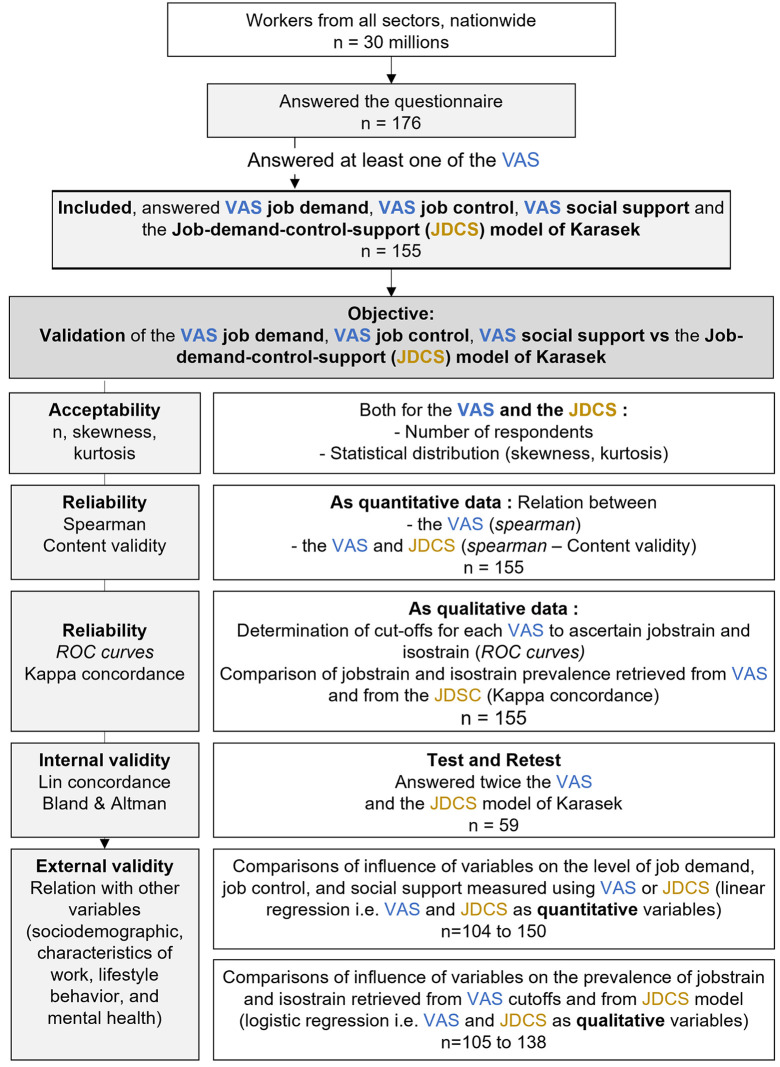
Flowchart of the analytical steps for the validation of the VAS demand, control, and support scales against the JDCS questionnaire, following COSMIN guidelines.

On the 176 workers who answered the questionnaire, we included 155 respondents (39.7 ± 11.5 years old, 75% women) who answered at least one of the VAS (test). Among the 155 respondents, 59 answered a second time after one week (re-test) (Fig 1). 131 records were complete on the 3 main dimensions of Kasarek’s model (job demand, job control and social support), both for VAS and JDCS questionnaire. Approximately half of the participants were executives or intellectual professional (48.2%) and had a level of education equal to or higher than Master’s level (49.9%). The participants who did only the test and those who did the test and retest did not differ in sociodemographic and lifestyle behavior, except for smoking (p = 0.045) (S1 Table in S1 File).

### Acceptability

**Fig 2:**
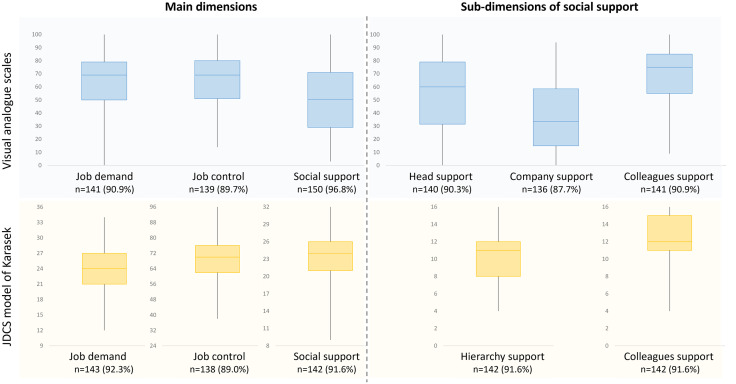
Acceptability – Score of the Karasek model items, as a quantitative variable, using the Visual Analog Scale (VAS) and the JDCS questionnaire. In the box plot (VAS in blue and Karasek in yellow), the lower and upper sides of the box are the lower and upper quartiles (Q1 and Q3). The box covers the interquartile interval (IQR), where 50% of the data is found. The horizontal line usually splits the box in two and is the median.

Response rate ranged from 87.7 to 96.8% for VAS, and from 89.0% to 92.3% for JDCS, with a tendency for higher response for VAS social support (p = 0.05). For the VAS (ranging from 0 to 100), mean score were 64.1 ± 21.4 for job demand, 65.6 ± 21.4 for job control, 51.6 ± 24.5 for job support (54.8 ± 28.3 for head support, 37.3 ± 26.6 for company support and 69.0 ± 22.0 for colleague support). For the JDCS, mean score were 24.1 ± 4.73 for job demand (possibly ranging from 9 to 36), 69.9 ± 11.2 for job control (from 24 to 96), and 23.3 ± 4.57 for social support (from 8 to 32) with 10.7 ± 2.91 for hierarchy support (from 4 to 16), and 12.6 ± 2.42 for colleague support (from 4 to 16). All dimensions of VAS and JDCS covered almost all possible values (Fig 2), with only the JDCS following a normal distribution. The distributions of VAS and JDCS, both as quantitative or as categorial variable, were symmetrical (moderate or good skewness values) and acceptable (most kurtosis values >2) (Table 1).

**Table 1:** Acceptability – Descriptive analysis of main outcome.

Variables (test)	Sample size N	Mean ± SD	Median [interquartile range]	Skewness	Kurtosis	Normality P-value
**Visual analog scale (VAS)**						
*VAS Job-demand*	141	**24.1 ± 4.73**	**69 [50; 79]**	**−0.856**	**3.363**	**<0.001**
*VAS Job-demand* <58	46 (%)	36.7 ± 17.5	43.5 [23; 50]	−0.739	2.311	
*VAS Job-demand* ≥ 58	95 (%)	77.4 ± 10.9	76 [69; 84]	0.589	2.449	
*VAS Job-control*	139	**65.6 ± 21.4**	**69 [51; 80]**	**−0.590**	**2.807**	**<0.001**
*VAS Job-control* <71.5	78 (%)	51.4 ± 17.1	55 [40; 67]	−0.768	2.377	
*VAS Job-control* ≥71.5	61 (%)	83.7 ± 9.42	83 [75; 91]	0.345	1.757	
*VAS Social Support*	150	**51.6 ± 24.5**	**50.5 [29; 71]**	**−0.129**	**1.983**	**0.002**
*VAS Social Support* <63.5	94 (%)	36.5 ± 17.1	37.5 [23; 50]	−0.768	2.377	
*VAS Social Support* ≥ 63.5	56 (%)	76.8 ± 9.57	75 [68; 83]	0.345	1.757	
**JDCS of Karasek**						
*Job-demand*	143	**24.1 ± 4.73**	**24 [21; 27]**	**−0.064**	**2.462**	0.96
*Job-demand* < 21	35 (%)	17.9 ± 2.06	19 [17; 20]	−0.984	3.353	
*Job-demand* ≥ 21	108 (%)	26.1 ± 3.44	26 [23; 28]	0.401	2.331	
*Job-control*	138	**69.9 ± 11.2**	**70 [62; 76]**	**−0.052**	**2.767**	0.88
*Job-control* <71	77 (%)	62.1 ± 7.13	64 [58; 68]	−0.928	3.375	
*Job-control* ≥71	61(%)	79.8 ± 6.49	78 [74; 84]	0.572	2.363	
*Social Support*	142	**23.3 ± 4.57**	**24 [21; 26]**	**−0.338**	**3.192**	0.13
*Social Support* <24	89 (%)	20.7 ± 3.35	21 [19; 24]	−1.149	4.070	
*Social Support* ≥ 24	53 (%)	27.8 ± 2.30	28 [26; 29]	0.624	2.263	

### Internal validity: Reliability (internal consistency)

Concerning *internal consistency*, job control and social support correlated between 0.28 and 0.36 for VAS, and between 0.41 to 0.50 for JDCS, with no relation with job demand (both for VAS and JDCS). The principal component analysis (PCA) confirmed that job demand (right bottom part of the PCA) is not related to job control nor social support (both in the left part of the PCA).

**Fig 3:**
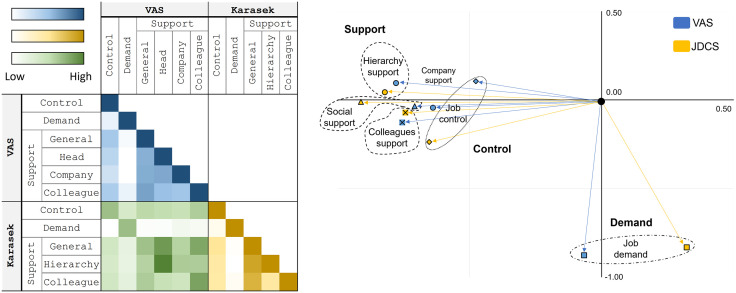
Internal consistency and content validity – Correlation between i) the Visual Analog Scale (VAS); ii) between the VAS and the JDCS questionnaire of Karasek items; iii) Principal component analysis. Factors that are located close together in the graph are well correlated. The PCA visually shows the proximity of VAS and JDCS for each sub-dimension.

For *content validity*, the correlations between VAS and JDCS were close to 0.50 for the main dimensions (0.51 for job control, 0.52 for job demand and 0.51 for social support), and between 0.33 and 0.81 for the subdimensions of social support (0.46 for hierarchy support, 0.66 for colleague’s support). The new VAS head support correlated with JDCS colleague’s support (r = 0.33), JDCS social support (r = 0.70), and JDCS hierarchy support (r = 0.81). The PCA confirmed that, for all dimensions and subdimensions, VAS and JDCS were located together. (Fig 3 and S1 Fig in S1 File).

### Internal validity: Cut-off determination and concordance

Using the cut-off of 20 for JDCS demand, ROC curve analysis retrieved a cut-off at **58** for **VAS job demand** with a satisfactory sensitivity (79%, 95 CI 70–86%), specificity (70%, 51–84), PPV (89%, 81–95%), and NPV (50%, 35–65%). The percentages of agreement between prevalence of workers with VAS job demand >58 and JDSC job demand >20 was 76.4%, with a kappa concordance coefficient of 0.42.

Using the cut-off of 71 for JDCS control, ROC curve analysis retrieved a cut-off at **71.5** for **VAS job control** with a satisfactory sensitivity (66%, 95 CI 53–78%), specificity (73%, 61–83), PPV (66%, 53–78%), and NPV (73%, 61–83%). The percentages of agreement between prevalence of workers with VAS job control >71.5 and JDSC job control >70 was 69.9%, with a kappa concordance coefficient of 0.39.

**Fig 4:**
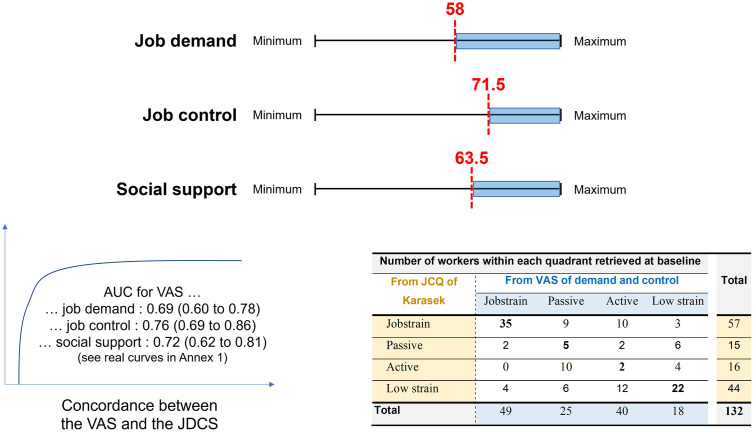
Internal validity – Threshold determination for occupational stress using the visual analog scale (VAS) of job demand, job control and social support (new tool), in reference to the JDCS questionnaire of Karasek (gold standard) – Receptive operator characteristics (ROC) curve; and concordance between the VAS and the JDCS quadrants.

Using the cut-off of 24 for JDCS social support, ROC curve analysis retrieved a cut-off at **63.5** for **VAS social support** with a satisfactory sensitivity (65%, 95 CI 51–78%), specificity (75%, 65–84), PPV (62%, 48–75%), and NPV (78%, 68–86%). The percentages of agreement between prevalence of workers with VAS social support >63.8 and JDSC social support >24 was 71.5%, with a kappa concordance coefficient of 0.40. We also calculated cut-off for subdimensions of VAS social support (Fig 4, Table 2, S2 Fig and S2 Table in S1 File).

**Table 2:** Internal validity – Cut-off determination for visual analog scales (VAS) and concordance with dimensions from the Job-Demand-Control Support (JDCS) questionnaire of Karasek.

Variables	Cut-off	TP/TN	FP/FN	Se (%)	Sp (%)	PPV (%)	NPV (%)	%	k
Job demand	58	84/23	10/23	78.5 [69.5–85.9]	69.7 [51.3–84.4]	89.4 [81.3–94.8]	50.0 [34.9–65.1]	76.4%	0.42
Job control	71.5	39/54	20/20	66.1 [52.6–77.9]	73.0 [61.4–82.6]	66.1 [52.6–77.9]	73.0 [61.4–82.6]	69.9%	0.39
Social support	63.5	34/64	21/18	65.4 [50.9–78.0]	75.3 [64.7–84.0]	61.8 [47.7–74.6]	78.0 [67.5–86.4]	71.5%	0.40
Company support	24.5	71/22	13/28	71.7 [61.8–80.3]	62.9 [44.9–78.5]	84.5 [75.0–91.5]	44.0 [30.0–58.7]	69.4%	0.30
Head support	38.5	31/92	5/10	90.2 [82.7–95.2]	86.1 [70.5–95.3]	94.8 [88.4–98.3]	75.6 [59.7–87.6]	89.1%	0.73
Colleague support	50.5	107/6	0/26	80.5 [72.7–86.8]	100 [54.1–100]	100.0 [96.6–100]	18.8 [7.2–36.4]	81.3%	0.26

TP: True positive; TN: True negative; FP: False positive; FN: False negative; Se: Sensibility (TP/(TP + FN)); Sp: Specificity (TN/(TN + FP); PPV: Positive predictive value (TP/(TP + FP); NPV: Negative predictive value (TN/(TN + FN); k: Kappa concordance coefficient.

### Internal validity: Concordance for job strain and isostrain

Considering quadrants from VAS and from the JDCS, 36.9 and 42.8% of workers were in high strain (“job strain”); and 21.2 and 20.5% were in isostrain, respectively. The Cohen concordance kappa was 0.43 [0.28–0.59] (Agreement = 72.7%) for job strain, and 0.41 [0.04–0.77] (Agreement = 80.0%) for isostrain (Table 3).

**Table 3:** Internal validity – Concordance between quadrants retrieved from visual analog scales (VAS) and quadrants retrieved from the Job Content Questionnaire (JCQ) of Karasek.

Number of workers within each quadrant retrieved at baseline									
From JDCS of Karasek	From VAS of demand and control					From JDCS of Karasek	From VAS of demand, control and social support		
	Jobstrain	Passive	Active	Low strain	Total		Jobstrain	Isostrain	Total
Jobstrain	**35**	9	10	3	57	Jobstrain	**4**	4	8
Passive	2	**5**	2	6	15				
Active	0	10	**2**	4	16	Isostrain	3	**24**	27
Low strain	4	6	12	**22**	44				
**Total**	49	25	40	18	**132**	**Total**	7	28	**35**

### Internal validity: Test–retest reproducibility

**Fig 5:**
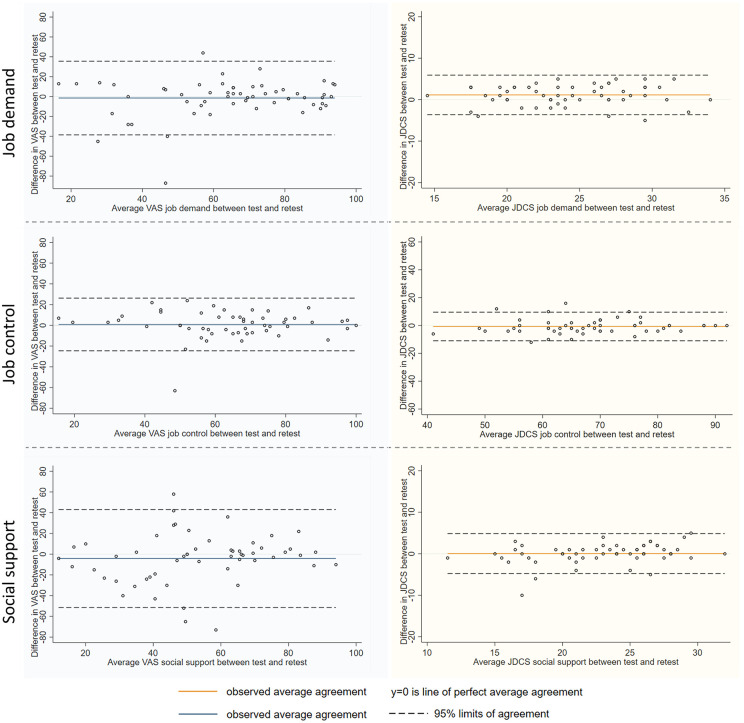
Test-retest reproducibility – Main dimensions agreement between test and retest measures for the visual analog scale (VAS) (new tool), and for the JDCS score (gold standard) – Bland et Altman plots. The horizontal axis represents the average score between test and retest for each dimension (VAS or JDCS). The vertical axis represents the difference between test and retest scores. The solid orange line shows the observed mean difference (average agreement), and the dashed lines indicate the 95% limits of agreement. The line at y = 0 represents perfect agreement between the two measurements.

Lin concordance correlation coefficient was 0.65 (0.50 to 0.80) for VAS job demand and 0.84 (0.76 to 0.91) for JDCS job demand, 0.79 (0.69 to 0.89) for VAS job control and 0.89 (0.83 to 0.95) for JDCS job control, and 0.46 (0.25 to 0.67) for VAS social support and 0.86 (0.80 to 0.93) for JDCS social support. For social support subdimensions, Lin concordance correlation coefficient was 0.68 (0.54 to 0.83) for VAS hierarchy and 0.87 (0.81 to 0.93) for JDCS hierarchy, 0.87 (0.80 to 0.93) for VAS colleagues and 0.78 (0.69 to 0.88) for JDCS colleagues, and 0.86 (0.79 to 0.93) for VAS head support. When VAS were categorized according to cut-offs, the Cohen concordance kappa was 0.56 (0.33 to 0.79) for VAS job demand (A = 81%) and 0.68 (0.46 to 0.89) for JDCS job demand (A = 88%), 0.68 (95 CI 0.48–0.89) for VAS job control (Agreement = 86%) and 0.64 (0.43 to 0.85) for JDCS job control (A = 83%), and 0.54 (0.32 to 0.77) for VAS social support (A = 78%) and 0.82 (0.67 to 0.97) for JDCS social support (A = 91%). For social support sub dimensions, the Cohen concordance kappa was 0.73 (0.55 to 0.91) for VAS hierarchy support (Agreement = 88%) and 0.81 (0.65 to 0.98) for JDCS hierarchy support (A = 93%), and 0.82 (0.66 to 0.99) for VAS colleague support (A = 93%) and 0.49 (0.11 to 1.00) JDCS colleague support (A = 96%), and 0.71 (0.52 to 0.91) for VAS head support (A = 88%). The test-retest reproducibility was also illustrated using Bland and Altman plots. Sensitivity analyses based on the time interval between test and retest also showed consistent reliability levels across both subgroups, confirming the temporal stability of the VAS measures. (Fig 5, S3 Fig and S3 Table in S1 File).

### External validity

**Fig 6:**
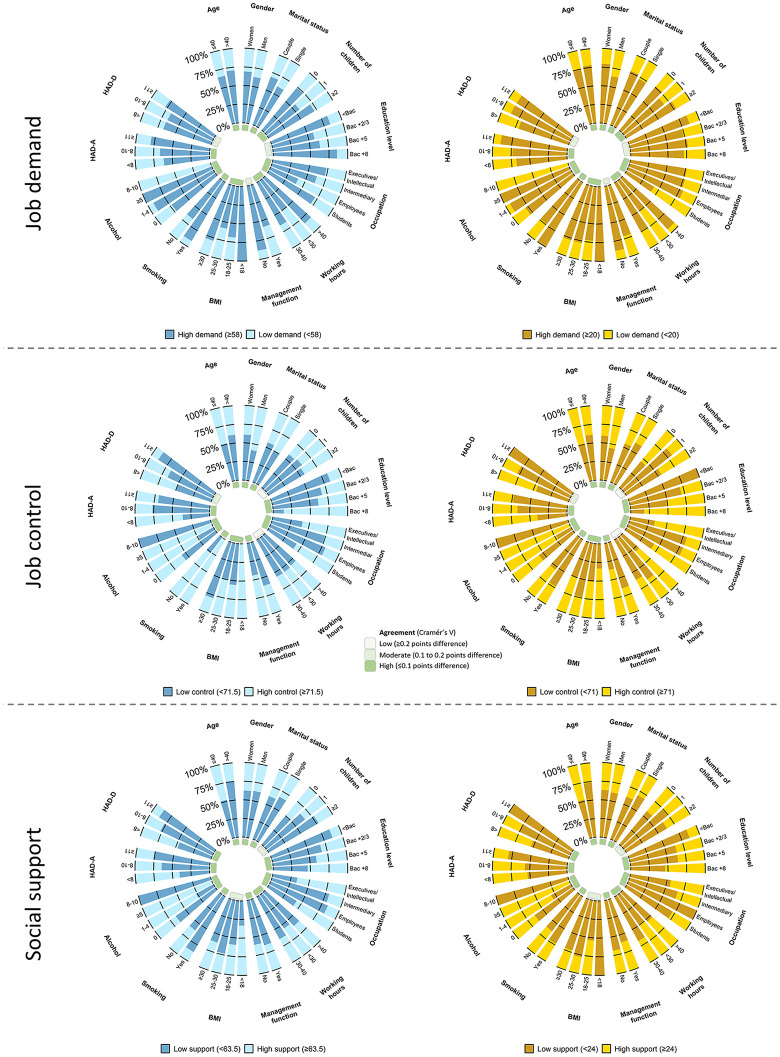
External validity – Relations between VAS and JDCS main items as categorical variables with secondary outcomes variables and agreements between measurement tools illustrated using a polar plot. The prevalence of high demand, low control and low support was compared between groups using a Chi² test. To quantify the strength of the association between secondary outcomes and each dimension, Cramer’s V was calculated. Agreement was considered low for ≥0.2 points difference, moderate for 0.1 to 0.2 points difference, and high for ≤0.1 points difference.

VAS and JDCS as **quantitative** variables, were similarly linked with all secondary outcomes, except a low agreement between VAS and the JDCS of Karasek for job demand and HAD, for job control and children/ management function, and for social support and education – with a visual representation using linear regression analyses (S4 Table in S1 File). VAS and JDCS as **qualitative** variables, were also similarly linked with all secondary outcomes (<0.10 difference on Cramer’s V), except a moderate agreement (0.1 to 0.2 points difference) for *job demand* and education/ management function, for *job control* and HAD-D, and for *social support* and children/ BMI; and a low agreement (≥0.2 points difference) for *job control* and working hours/ children (S5 Table in S1 File) – with a visual representation using polar plots (Fig 6) and logistic regression analyses (S4 Fig in S1 File). External validity for subdimensions and quadrants are presented in S6 et S7 Tables in S1 File.

**Fig 7:**
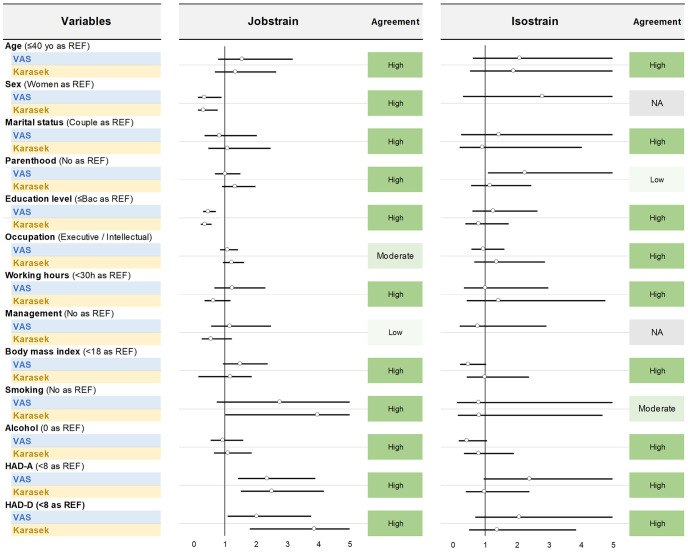
External validity – Relation between VAS and JDCS (treated as Jobstrain and Isostrain) with secondary outcome variables, and agreement between measurement tools illustrated using a forest plot. The effect of each variable on the risk of Jobstrain/ Isostrain is represented by a dot on a horizontal line. The dots represent the risk of Jobstrain or Isostrain (odds ratio) for each variable, and the line around the dots represent their 95% confidence interval (95 CI). The vertical line represents the null estimate (with a value of 1). Odds ratio with horizontal lines that do not cross the vertical line are significant. Significant variables with an odds ratio <1 are protective factors and those with an odds ratio >1 are risk factors. REF: Reference, i.e., the reference for group comparisons.

As well, prevalence of jobstrain and isostrain calculated using VAS or the JDCS were similarly linked with all secondary outcomes, except a moderate agreement for jobstrain and occupation/ HAD-D, and for isostrain and children/ education/ BMI/ alcohol, and a low agreement for isostrain and working hours/ management function (Table 4) – with a visual representation using polar plots (S5 Fig in S1 File) and logistic regression analyses (Fig 7).

**Table 4:** External validity – Relation between VAS and JDCS (treated as Jobstrain and Isostrain) with secondary outcomes variables and agreements between measurement tools.

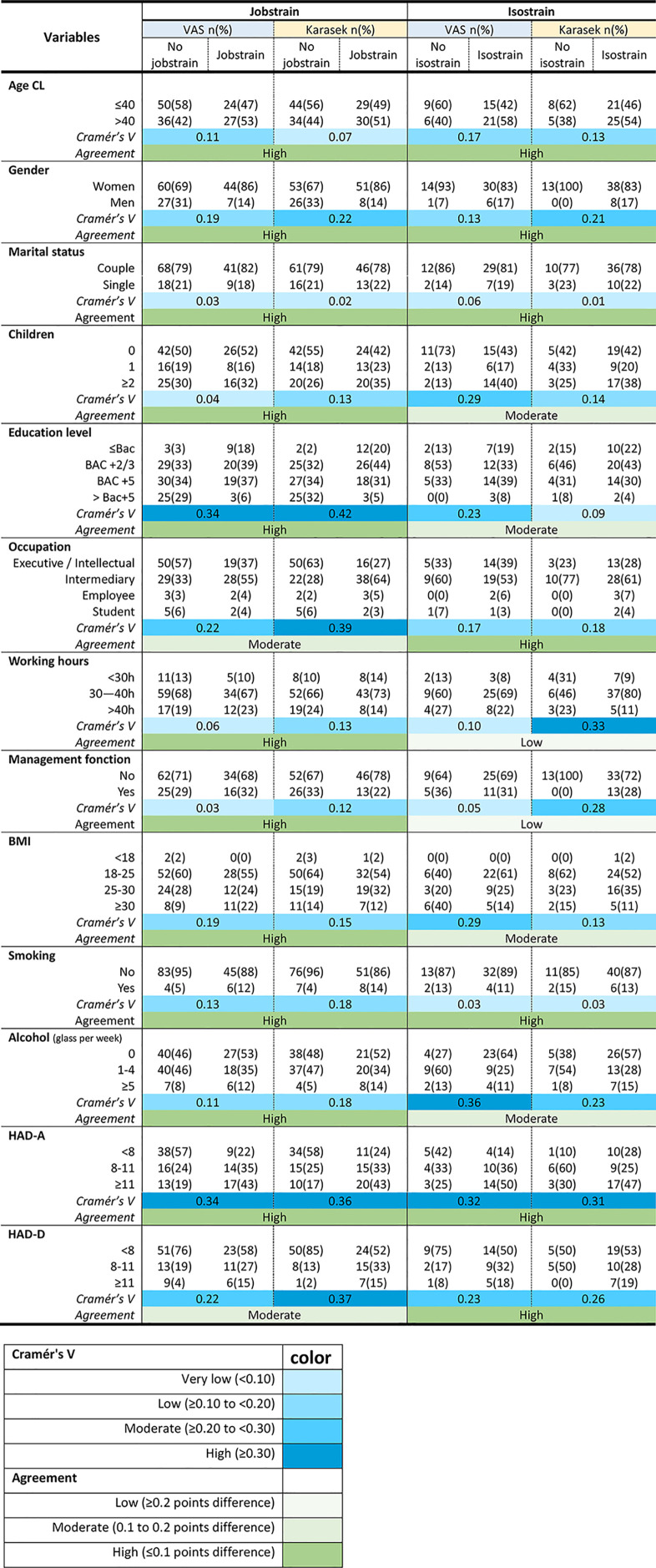

## Discussion

This study enabled the validation of visual analogue scales to assess job demand, job control and social support in the workplace, emphasizing their acceptability, internal validity, reproducibility, and external validity.

### Acceptability: Psychometrics properties

Occupational stress – i.e., jobstrain – has emerged as a significant concern in modern workplaces, with far-reaching implications for both individuals and organizations. At the individual level, prolonged exposure to occupational stressors was found to be associated with a range of negative health outcomes, including increased risk of cardiovascular diseases, mental health disorders, and burnout [33]. These health issues not only diminish the quality of life for affected employees but also lead to decreased job satisfaction and reduced productivity [34]. From an organizational perspective, the consequences of occupational stress are equally concerning. Companies experiencing high levels of employee stress face increased absenteeism, higher turnover rates, and diminished overall performance [35]. The large impact of occupational stress is a major public health problem that needs to be tackled. Occupational physicians are on the front line in monitoring workplace health and can play a central role in detecting psychosocial risks [36]. Unfortunately, occupational physicians generally have limited time to address a wide range of occupational risks on a large number of workers [37]. More specifically, even if the JDCS model of Karasek is the gold standard for assessing occupational stress at work, its length makes it challenging for occupational practitioners to incorporate into daily medical procedures. Developing quick and straightforward screening tools is essential in the era of preventive medicine [38]. VAS are already frequently used by occupational physicians [22,39]. These scales are easy to implement and understand, quick to administer, and allow for systematic and standardized use in routine practice. The response rate to the VAS in our study was excellent, ranging from 87.7 to 96.8% for all VAS and 89.0% to 92.3% for JDCS and all dimensions covered almost all possible values.

### Internal validity

Cut-offs for VAS of job control, job demand and social support were retrieved respectively at 71.5, 58 and 63.5. Easier-to-remember cut-offs could be proposed at 70, 60 and 60, similarly to cut-offs for stress, anxiety, or depression, that were rounded at 60 or 70 [28,40]. These cut-offs could help physicians to detect and treat patients in jobstrain/ isostrain. According to this study, VAS developed to assess occupational stress through job demand, job control and social support items seems to be a reliable and precise instrument to conduct large scale screening on occupational health services. Lin’s concordance coefficient ranged from 0.46 to 0.87, generally indicating good to excellent agreement, although some dimensions showed weaker agreement compared to the JDCS and Karasek measures [41]. We found significant correlations between each JDCS items and its corresponding VAS. The cut-off for job demand showed high sensitivity (78.5%) but moderate specificity (69.7%), with a kappa coefficient of 0.42. PPV is high (89.4%), but NPV is low (50.0%), meaning it effectively identifies high job demand but requires caution regarding false negatives. Both for the cut-off for job control and social support, moderate sensitivity and specificity were found, with a kappa coefficient around 0.40. PPV and NPV were also moderate. From a clinical perspective, a kappa of about 0.40 represents moderate agreement with the JDCS, which, although imperfect, is acceptable for screening purposes in occupational health. It suggests that the VAS can correctly identify most employees at risk of high job demand, low control, or low social support, while some cases may remain undetected. Thus, the VAS provides a practical and time-efficient tool for initial assessment, to be followed by the full JCQ when results exceed the proposed cut-offs or when more detailed evaluation is required.

### External validity

The generalizability of the new VAS is ensured by its external validity. For this, we studied the relationship between the VAS and the JDCS and the secondary outcomes. There were linked in the same way with the secondary outcomes in 77% of cases for demand and control, both as continuous and categorical variable (10 high agreement out of 13 variables). For social support, the results were similar at 69% (9 variables) categorically versus 85% (11 variables) continuously between the two measurement tools. For both quadrants and jobstrain assessment, VAS and JDCS were similarly related to secondary variables in 85% of cases (11 high agreement on 13 variables). Finally, for isostrain, we found 54% of high agreement (7 variables) between measurement tools. As found in the literature, a higher number of hours worked per week and management function were associated with higher job demand in both the VAS and the JDCS, supporting the idea that working time and managerial duties are key determinants of perceived job demands [42]. Similarly, education level, occupation, and psychological distress (HAD) were associated with job control in both models. This aligns with previous research showing that higher education and executives’ function often provide individuals greater autonomy at work, while lower perceived control is linked to higher psychological distress [43]. However, the number of children and working hours were associated with job control in the VAS but not in the JDCS, suggesting that VAS may be more sensitive to certain contextual or lifestyle factors, although this finding should be interpreted with caution. For social support, both the VAS and JDCS assessments revealed significant associations with age and psychological distress (HAD). This finding is consistent with the literature, suggesting that social support at work may vary with age and that lower perceived support is linked to higher levels of distress [44,45]. Finally, considering jobstrain, both the VAS and JDCS assessments revealed associations with education levels and HAD [46].

### Limitations

There are several limitations on the study. When compared to other research that used questionnaires in French populations, the response rate may seem low [14]. However, we followed the COSMIN recommendations to determine the sample size and the number of respondents was sufficient to carry out statistical analyses [31]. Furthermore, the voluntary nature of participation may have introduced a self-selection bias, as workers who are more aware of or interested in psychosocial risks might have been more likely to respond. Consequently, women and individuals with management functions were overrepresented, precluding the generalizability of our findings, as stress perception and coping strategies may differ across gender and occupational levels [47–49]. This pattern is consistent with a well-documented trend in occupational and psychosocial research, where women and highly educated individuals tend to participate more frequently in voluntary surveys. Women may be more likely to engage due to higher health awareness, greater willingness to report stress, or sociocultural norms that make expressing psychological distress more acceptable [50–52]. Similarly, executives and highly educated participants may be overrepresented because of greater familiarity with online surveys, stronger identification with the research topic, or perceived value of participation [53]. Together, these factors underscore the need for future studies to recruit more diverse samples, including male workers and underrepresented occupational groups, to validate the robustness of the VAS measures across different subpopulations. Regarding measuring tools, VAS may seem too superficial since they assess a dimension through one item when a questionnaire assesses it through several items (e.g., Karasek 9 for demand, 9 for control and 8 for social support). However, VAS can limit the loss of attention and concentration associated with the length and complexity of questionnaires [54]. Finally, the test–retest reliability was assessed over a one-week interval, a duration considered long enough to minimize recall or memory effects, yet short enough to reduce the likelihood of genuine changes in participants’ work environments or mental health. According to methodological recommendations, the interval between repeated administrations should strike this balance, sufficiently long to prevent recall, but sufficiently short to avoid clinical or contextual change [29]. Thus, while the chosen period may vary depending on the construct assessed, a one to two week interval is generally considered appropriate, provided that it is clearly described and justified [30]. Future studies should aim to replicate these findings in more diverse and representative samples and assess the long-term stability of the VAS measures.

## Conclusion

Even though they are not as effective as the job-demand-control-support (JDCS) model of Karasek, the VAS is a quick and easy tool for screening individuals with major work-related stress. Thus, primary prevention and diagnosis can be achieved using VAS in daily clinical practice. Based on our findings, we propose cut-offs of 60, 70, and 60 for VAS job demand, VAS job control, and VAS social support, respectively. These thresholds can help occupational physicians identify workers at higher risk. However, we advise the use of the JDCS to be more specific and discriminating for workers with VAS values over the cut-offs.

## Supporting information

S1 File Synthesis of all figures and statistics. (DOCX)

S2 File Questionnaire. (DOCX)

S3 File Database. (XLSX)

## References

[1] Anderson DL. Organization development: the process of leading organizational change. 2013. pp. 392.

[2] Dubois C-A, Bentein K, Mansour JB, Gilbert F, Bédard J-L. Why some employees adopt or resist reorganization of work practices in health care: associations between perceived loss of resources, burnout, and attitudes to change. Int J Environ Res Public Health. 2013;11(1):187–201. doi: 10.3390/ijerph110100187 24362547 PMC3924440

[3] Karasek R, Brisson C, Kawakami N, Houtman I, Bongers P, Amick B. The Job Content Questionnaire (JCQ): an instrument for internationally comparative assessments of psychosocial job characteristics. J Occup Health Psychol. 1998;3(4):322–55. doi: 10.1037//1076-8998.3.4.322 9805280

[4] Backé E-M, Seidler A, Latza U, Rossnagel K, Schumann B. The role of psychosocial stress at work for the development of cardiovascular diseases: a systematic review. Int Arch Occup Environ Health. 2012;85(1):67–79. doi: 10.1007/s00420-011-0643-6 21584721 PMC3249533

[5] Silva-Junior JSD, Fischer FM. Long-term sickness absence due to mental disorders is associated with individual features and psychosocial work conditions. PLoS One. 2014;9(12):e115885. doi: 10.1371/journal.pone.0115885 25531900 PMC4274157

[6] Cohen S, Janicki-Deverts D, Miller GE. Psychological stress and disease. JAMA. 2007;298(14):1685–7. doi: 10.1001/jama.298.14.1685 17925521

[7] Ruotsalainen JH, Verbeek JH, Marine A, Serra C. Preventing occupational stress in healthcare workers. Cochrane Database Syst Rev. 2014;13(11). 10.1002/14651858.CD002892.pub4 25482522

[8] Karasek RA. Job demands, job decision latitude, and mental strain: implications for job redesign. Admin Sci Q. 1979;24(2):285. doi: 10.2307/2392498

[9] Theorell T, Karasek RA. Current issues relating to psychosocial job strain and cardiovascular disease research. J Occup Health Psychol. 1996;1(1):9–26. doi: 10.1037//1076-8998.1.1.9 9547038

[10] Pisanti R, van der Doef M, Maes S, Lazzari D, Bertini M. Job characteristics, organizational conditions, and distress/well-being among Italian and Dutch nurses: a cross-national comparison. Int J Nurs Stud. 2011;48(7):829–37. doi: 10.1016/j.ijnurstu.2010.12.006 21257172

[11] Karasek R, Baker D, Marxer F, Ahlbom A, Theorell T. Job decision latitude, job demands, and cardiovascular disease: a prospective study of Swedish men. Am J Public Health. 1981;71(7):694–705. doi: 10.2105/ajph.71.7.694 7246835 PMC1619770

[12] Niedhammer I, Chastang J-F, Levy D, David S, Degioanni S, Theorell T. Study of the validity of a job-exposure matrix for psychosocial work factors: results from the national French SUMER survey. Int Arch Occup Environ Health. 2008;82(1):87–97. doi: 10.1007/s00420-008-0311-7 18327603

[13] Niedhammer I, Chastang JF, Gendrey L, David S, Degioanni S. Psychometric properties of the French version of Karasek’s “Job Content Questionnaire” and its scales measuring psychological pressures, decisional latitude and social support: the results of the SUMER. Sante Publique. 2006;18(3):413–27. doi: 10.3917/spub.063.0413 17094683

[14] Dutheil F, Pereira B, Moustafa F, Naughton G, Lesage F-X, Lambert C. At-risk and intervention thresholds of occupational stress using a visual analogue scale. PLoS One. 2017;12(6):e0178948. doi: 10.1371/journal.pone.0178948 28586383 PMC5460813

[15] Li J, Riedel N, Barrech A, Herr RM, Aust B, Mortl K. Long-term effectiveness of a stress management intervention at work: a 9-year follow-up study based on a randomized wait-list controlled trial in male managers. Biomed Res Int. 2017;2017(10):18. doi: 10.1155/2017/2853813 PMC5664277 29181392

[16] Carlsson F, Merlo J, Lindström M, Ostergren P-O, Lithman T. Representativity of a postal public health questionnaire survey in Sweden, with special reference to ethnic differences in participation. Scand J Public Health. 2006;34(2):132–9. doi: 10.1080/14034940510032284 16581705

[17] Reilly EE, Brown TA, Wierenga CE. Evaluating patterns of inconsistent and missing data on the eating disorders examination-questionnaire in a sample of treatment-seeking adults and adolescents. Eat Disord. 2021;29(5):550–9. doi: 10.1080/10640266.2019.1695453 31775572

[18] Cohen S, Kamarck T, Mermelstein R. A global measure of perceived stress. J Health Soc Behav. 1983;24(4):385. doi: 10.2307/2136404 6668417

[19] Arapovic-Johansson B, Wåhlin C, Kwak L, Björklund C, Jensen I. Work-related stress assessed by a text message single-item stress question. Occup Med (Lond). 2017;67(8):601–8. doi: 10.1093/occmed/kqx111 29016877 PMC5927000

[20] Lesage FX, Berjot S. Validity of occupational stress assessment using a visual analogue scale. Occup Med (Lond). 2011;61(6):434–6. doi: 10.1093/occmed/kqr037 21505089

[21] Lesage F-X, Berjot S, Deschamps F. Clinical stress assessment using a visual analogue scale. Occup Med (Lond). 2012;62(8):600–5. doi: 10.1093/occmed/kqs140 22965867

[22] Boonstra AM, Schiphorst Preuper HR, Reneman MF, Posthumus JB, Stewart RE. Reliability and validity of the visual analogue scale for disability in patients with chronic musculoskeletal pain. Int J Rehabil Res. 2008;31(2):165–9. doi: 10.1097/MRR.0b013e3282fc0f93 18467932

[23] Ratinaud MC, Chamoux A, Glace B, Coudeyre E. Job satisfaction evaluation in low back pain: a literature review and tools appraisal. Ann Phys Rehabil Med. 2013;56(6):465–81. doi: 10.1016/j.rehab.2013.06.006 23928031

[24] Eisenberger R, Huntington R, Hutchison S, Sowa D. Perceived organizational support. J Appl Psychol. 1986;71(3):500–7. doi: 10.1037/0021-9010.71.3.500

[25] Kurtessis JN, Eisenberger R, Ford MT, Buffardi LC, Stewart KA, Adis CS. Perceived organizational support: a meta-analytic evaluation of organizational support theory. J Manage. 2015;43(6):1854–84. doi: 10.1177/0149206315575554

[26] Harris PA, Taylor R, Minor BL, Elliott V, Fernandez M, O’Neal L, et al. The REDCap consortium: building an international community of software platform partners. J Biomed Inform. 2019;95:103208. doi: 10.1016/j.jbi.2019.103208 31078660 PMC7254481

[27] Harris PA, Taylor R, Thielke R, Payne J, Gonzalez N, Conde JG. Research electronic data capture (REDCap)--a metadata-driven methodology and workflow process for providing translational research informatics support. J Biomed Inform. 2009;42(2):377–81. doi: 10.1016/j.jbi.2008.08.010 18929686 PMC2700030

[28] Dutheil F, Palgen C, Brousse G, Cornet T, Mermillod M, Lakbar I, et al. Validation of visual analog scales of mood and anxiety at the workplace. PLoS One. 2024;19(12):e0316159. doi: 10.1371/journal.pone.0316159 39739967 PMC11687878

[29] Marx RG, Menezes A, Horovitz L, Jones EC, Warren RF. A comparison of two time intervals for test-retest reliability of health status instruments. J Clin Epidemiol. 2003;56(8):730–5. doi: 10.1016/s0895-4356(03)00084-2 12954464

[30] Terwee CB, Bot SDM, de Boer MR, van der Windt DAWM, Knol DL, Dekker J, et al. Quality criteria were proposed for measurement properties of health status questionnaires. J Clin Epidemiol. 2007;60(1):34–42. doi: 10.1016/j.jclinepi.2006.03.012 17161752

[31] Mokkink LB, Terwee CB, Knol DL, Stratford PW, Alonso J, Patrick DL, et al. Protocol of the COSMIN study: COnsensus-based Standards for the selection of health Measurement INstruments. BMC Med Res Methodol. 2006;6:2. doi: 10.1186/1471-2288-6-2 16433905 PMC1368990

[32] Knell G, Gabriel KP, Businelle MS, Shuval K, Wetter DW, Kendzor DE. Ecological momentary assessment of physical activity: validation study. J Med Internet Res. 2017;19(7):e253. doi: 10.2196/jmir.7602 28720556 PMC5539388

[33] Coelho LG, Costa PRDF, Kinra S, Pitangueira JCD, Lira CRDN, Akutsu RDCC. The influence of occupational stress on workers’ health: systematic review and meta-analysis. Res Soc Dev. 2022;11(3):e23111326449. doi: 10.33448/rsd-v11i3.26449

[34] Ji J, He B, Gong S, Sheng M, Ruan X. Network analysis of occupational stress and job satisfaction among radiologists. Front Public Health. 2024;12:1411688. doi: 10.3389/fpubh.2024.1411688 38952733 PMC11215115

[35] Salama W, Abdou AH, Mohamed SAK, Shehata HS. Impact of work stress and job burnout on turnover intentions among hotel employees. Int J Environ Res Public Health. 2022;19(15):9724. doi: 10.3390/ijerph19159724 35955081 PMC9368148

[36] Jain A, Hassard J, Leka S, Di Tecco C, Iavicoli S. The role of occupational health services in psychosocial risk management and the promotion of mental health and well-being at work. Int J Environ Res Public Health. 2021;18(7):3632. doi: 10.3390/ijerph18073632 33807352 PMC8036601

[37] Eisch E, Kuper P, Lindert L, Choi K-EA. Working conditions of occupational physicians-a scoping review. Int J Environ Res Public Health. 2022;19(10):6222. doi: 10.3390/ijerph19106222 35627762 PMC9141582

[38] Dutheil F, Pereira B, Bouillon-Minois J-B, Clinchamps M, Brousses G, Dewavrin S, et al. Validation of visual analogue scales of job demand and job control at the workplace: a cross-sectional study. BMJ Open. 2022;12(3):e046403. doi: 10.1136/bmjopen-2020-046403 35301199 PMC8932271

[39] Lesage F-X, Berjot S, Deschamps F. Clinical stress assessment using a visual analogue scale. Occup Med (Lond). 2012;62(8):600–5. doi: 10.1093/occmed/kqs140 22965867

[40] Lesage FX, Berjot S. Validity of occupational stress assessment using a visual analogue scale. Occup Med (Lond). 2011;61(6):434–6. doi: 10.1093/occmed/kqr037 21505089

[41] Lin LI. A concordance correlation coefficient to evaluate reproducibility. Biometrics. 1989;45(1):255–68. doi: 10.2307/2532051 2720055

[42] Boucher F, Dextras-Gauthier J, Gilbert M-H, Fournier P-S, Dima J. One down, fifty to go: managers’ perceptions of their workload and how they cope with it to maintain their psychological health. Front Psychol. 2024;14:1336560. doi: 10.3389/fpsyg.2023.1336560 38374933 PMC10876056

[43] Moreno-Pimentel AG, Meneses Monroy A, Martín-Casas P, Zaragoza-García I, Girón-Daviña P. Impact of social and occupational factors over job control. Med Lav. 2019;110(3):226–33. doi: 10.23749/mdl.v110i3.7925 31268429 PMC7812539

[44] Conte KP, Schure MB, Goins RT. Correlates of social support in older American Indians: the Native Elder Care Study. Aging Ment Health. 2015;19(9):835–43. doi: 10.1080/13607863.2014.967171 25322933 PMC5338610

[45] Ozaki K, Motohashi Y, Kaneko Y, Fujita K. Association between psychological distress and a sense of contribution to society in the workplace. BMC Public Health. 2012;12:253. doi: 10.1186/1471-2458-12-253 22463500 PMC3369557

[46] Stansfeld S, Candy B. Psychosocial work environment and mental health--a meta-analytic review. Scand J Work Environ Health. 2006;32(6):443–62. doi: 10.5271/sjweh.1050 17173201

[47] Kelly MM, Tyrka AR, Price LH, Carpenter LL. Sex differences in the use of coping strategies: predictors of anxiety and depressive symptoms. Depress Anxiety. 2008;25(10):839–46. doi: 10.1002/da.20341 17603810 PMC4469465

[48] Matud MP. Gender differences in stress and coping styles. Pers Individual Diff. 2004;37(7):1401–15. doi: 10.1016/j.paid.2004.01.010

[49] Verma R, Balhara YPS, Gupta CS. Gender differences in stress response: role of developmental and biological determinants. Ind Psychiatry J. 2011;20(1):4–10. doi: 10.4103/0972-6748.98407 22969173 PMC3425245

[50] Couarraze S, Decormeille G, Delamarre L, Marhar F, Gbaglo K, Avilès Dorlhiac R, et al. Impact of teleworking on work-related and home-related stress at during the first global lockdown-the international COVISTRESS study. Brain Behav. 2025;15(6):e70592. doi: 10.1002/brb3.70592 40444578 PMC12123451

[51] Couarraze S, Delamarre L, Marhar F, Quach B, Jiao J, Avilés Dorlhiac R, et al. The major worldwide stress of healthcare professionals during the first wave of the COVID-19 pandemic - the international COVISTRESS survey. PLoS One. 2021;16(10):e0257840. doi: 10.1371/journal.pone.0257840 34614016 PMC8494302

[52] Dutheil F, Saint-Arroman C, Clinchamps M, Flaudias V, Fantini ML, Pereira B, et al. Influence of socio-demographic, occupational and lifestyle variables on sleep time. Nat Sci Sleep. 2025;17:195–210. doi: 10.2147/NSS.S495455 39963101 PMC11832214

[53] Reinikainen J, Tolonen H, Borodulin K, Härkänen T, Jousilahti P, Karvanen J, et al. Participation rates by educational levels have diverged during 25 years in Finnish health examination surveys. Eur J Public Health. 2018;28(2):237–43. doi: 10.1093/eurpub/ckx151 29036286

[54] Rolstad S, Adler J, Rydén A. Response burden and questionnaire length: is shorter better? A review and meta-analysis. Value Health. 2011;14(8):1101–8. doi: 10.1016/j.jval.2011.06.003 22152180

