# Supplementary material for: Validation of visual analogue scales to assess occupational stress compared to the Karasek questionnaire: A cross sectional study
Source: PLoS One. 2026 Feb 10;21(2):e0340209. doi: 10.1371/journal.pone.0340209 (PMC12890140; doi:10.1371/journal.pone.0340209)
Supplement: S1 File — (DOCX) [file pone.0340209.s001.docx]

**Supplementary materials**

Synthesizing all figures and statistics

[S1 Supporting Information. JCQ and VAS description and scoring 3](#_Toc212113066)

[Fig 1. Flowchart of the analytical steps for the validation of the VAS demand, control, and support scales against the JDCS questionnaire, following COSMIN guidelines 4](#_Toc212113067)

[S1 Table. Characteristics of participants 5](#_Toc212113068)

[Fig 2. Acceptability – Score of the Karasek model items, as a quantitative variable, using the Visual Analog Scale (VAS) and the Job-Demand-Control Support (JDCS) questionnaire of Karasek 6](#_Toc212113069)

[Table 1. Acceptability – Descriptive analysis of main outcome 7](#_Toc212113070)

[S1 Fig. Internal consistency and content validity – Correlation between i) the Visual Analog Scale (VAS); ii) between the VAS and the Job-Demand-Control Support (JDCS) questionnaire of Karasek; iii) Principal component analysis 8](#_Toc212113071)

[Table 2. Internal validity – Cut-off determination for visual analog scales (VAS) and concordance with dimensions from the Job-Demand-Control Support (JDCS) questionnaire of Karasek 9](#_Toc212113072)

[S2 Table. Internal validity – Sensitivity analysis on rounded cut-off for visual analog scales (VAS) and concordance with dimensions from the Job-Demand-Control Support (JDCS) questionnaire of Karasek 10](#_Toc212113073)

[S2 Fig. Internal validity – Threshold determination for occupational stress using the visual analog scale (VAS) of job demand, job control and social support (new tool), in reference to the JDCS questionnaire of Karasek (gold standard) – Receptive operator characteristics (ROC) curve; and concordance between the VAS and the JDCS quadrants 11](#_Toc212113074)

[Fig 5. Test-retest reproducibility – Main dimensions agreement between test and retest measures for the visual analog scale (VAS) (new tool), and for the JDCS score (gold standard) – Bland et Altman plots 12](#_Toc212113075)

[S3 Fig. Test-retest reproducibility – Support sub-dimensions agreement between test and retest measures for the visual analog scale (VAS) (new tool), and for the JDCS score (gold standard) – Bland et Altman plots 13](#_Toc212113076)

[S3 Table. Test-retest reproducibility – Sensitivity analysis comparing test–retest reliability coefficients for participants who completed the retest within 7 days (n = 28) versus after 7 days (n = 27) 14](#_Toc212113077)

[S4 Table. External validity – Relation of VAS and JDCS main items as continuous variables with secondary outcomes and agreements between measurement tools 15](#_Toc212113078)

[S5 Table. External validity – Relations between VAS and JDCS main items as categorical variables with secondary outcomes variables and agreements between measurement tools 17](#_Toc212113079)

[Fig 6. External validity – Relations between VAS and JDCS main items as categorical variables with secondary outcomes variables and agreements between measurement tools illustrated using a polar plot 19](#_Toc212113080)

[S4 Fig. External validity – Relations between VAS and JDCS main items as continuous variables with secondary outcomes variables and agreements between measurement tools illustrated using a forest plot 20](#_Toc212113081)

[S6 Table. External validity – Relations between VAS and JDCS sub-dimensions items according to cut-offs with other variables and agreements between measurement tools 21](#_Toc212113082)

[S7 Table. External validity – Relations between VAS and JDCS treated as quadrants with secondary outcomes variables and agreements between measurement tools 23](#_Toc212113083)

[Table 4. External validity – Relation between VAS and JDCS (treated as Jobstrain and Isostrain) with secondary outcomes variables and agreements between measurement tools 25](#_Toc212113084)

[S5 Fig. External validity – Relation between VAS and JDCS (treated as Jobstrain and Isostrain) with secondary outcome variables illustrated using a polar plot 27](#_Toc212113085)

[Fig 7. External validity – Relation between VAS and JDCS (treated as Jobstrain and Isostrain) with secondary outcome variables, and agreement between measurement tools illustrated using a forest plot 28](#_Toc212113086)

# S1 Supporting Information. JCQ and VAS description and scoring

The **Job-Content Questionnaire (JCQ),** derived from the Job Demand-Control-Support (JDCS) model, is a 26-item instrument assessing three main dimensions: **job demand, job control,** and **social support.** All items (Q1–Q26) are scored on a 4-points Likert scale (from 1 to 4).

According to Karasek, the score for each dimension was determined using these equations:

- Job demand = Q10 + Q11 + Q12 + (5-Q13) + Q14 + Q15 + Q16 + Q17 + Q18
- Job control = 4*Q4 + 4*(5-Q6) + 4*(Q8) + 2*(5-Q2) + 2*(Q5) + 2*(Q7) + 2*(Q1) + 2*(Q3) + 2*(Q9)
- Social support = Q19+Q20+Q21+Q22+Q23+Q24+Q25+Q26

The quadrants of Karasek were determined as follow, based on the standard approach used in previous studies with the French version of the JCQ:

- “Job strain” = demand score >20 and control score <71
- “Passive” = demand score <20 and control score <71)
- “Active” for demand score >20 and control score <71
- “Low strain” = demand score <20 and control score >71.
- “Isostrain” = job strain situation and a social support score <24.

**Visual analogue scales**: Each dimension of the Job Demand-Control-Support (JDCS) model was assessed using a 100-mm Visual Analogue Scale (VAS) ranging from 0 (“minimum”) to 100 (“maximum”). Participants were asked to answer the following VAS:

- **Job demand :** « Dans le cadre de votre travail, quel est votre niveau de demande psychologique au travail (charge de travail) ? »
- **Job control :** « Dans le cadre de votre travail, quel est votre niveau de latitude décisionnelle / autonomie au travail ? »
- **Support from colleagues :** « Dans le cadre de votre travail, quel est votre niveau de soutien de vos collègues ?”
- **Support from direct supervisor (head support)**: « Dans le cadre de votre travail, quel est votre niveau de soutien de votre chef (supérieur hiérarchique) ? »
- **Support from the company / organization (institutional support) :** « Dans le cadre de votre travail, quel est votre niveau de soutien de votre hiérarchie (institution/entreprise) ? »
- **General social support :** « Dans le cadre de votre travail, quel est votre niveau de soutien social en général ? »

Higher scores on job demand are associated with greater risk, whereas higher scores on job control and support are associated with protective effects.

# Fig 1. Flowchart of the analytical steps for the validation of the VAS demand, control, and support scales against the JDCS questionnaire, following COSMIN guidelines

# S1 Table. Characteristics of participants

| **Characteristics of participants** | **n** | **Test** | **Retest** | ***P-value*** |
| --- | --- | --- | --- | --- |
| Age |  |  |  |  |
| Years mean ± SD |  | 39.7±11.5 | 39.1±10.3 | 0.70 |
| ≤40, n (%) | 89 | 55 (51.4%) | 34 (57.6%) | 0.44 |
| >40, n (%) | 77 | 52 (48.6%) | 25 (42.4%) |  |
| Sex, n (%) |  |  |  |  |
| Women | 123 | 76 (72.4 %) | 47 (79.7%) | 0.30 |
| Men | 41 | 29 (27.6%) | 12 (20.3%) |  |
| Marital Status, n (%) |  |  |  |  |
| Couple | 128 | 79 (76.7%) | 49 (83.1%) | 0.34 |
| Single | 34 | 24 (23.3%) | 10 (17.0%) |  |
| Children, n (%) |  |  |  |  |
| 0 | 80 | 53 (51.0%) | 27 (47.4%) | 0.90 |
| 1 | 33 | 21 (20.2%) | 12 (21.0%) |  |
| ≥2 | 48 | 30 (28.8%) | 18 (31.6%) |  |
| Education level, n (%) |  |  |  |  |
| <Bac | 18 | 10 (9.26%) | 8 (13.6%) | 0.42 |
| Bac +2/3 | 62 | 43 (39.8%) | 19 (32.2%) |  |
| Bac +5 | 56 | 38 (35.2%) | 18 (30.5%) |  |
| Bac +8 | 31 | 17 (15.7%) | 14 (23.7%) |  |
| Occupation, n (%) |  |  |  |  |
| Executives / Intellectual | 79 | 52 (48.2%) | 27 (45.8%) | 0.96 |
| Intermediary | 73 | 46 (42.6%) | 27 (45.8%) |  |
| Employee | 5 | 3 (2.78%) | 2 (3.39%) |  |
| Students | 10 | 7 (6.48%) | 3 (5.08%) |  |
| Work hours, n (%) |  |  |  |  |
| <30 | 21 | 16 (14.8%) | 5 (8.47%) | 0.30 |
| 30-40 | 112 | 73 (67.6%) | 39 (66.1%) |  |
| >40 | 34 | 19 (17.6%) | 15 (25.4%) |  |
| Management, n (%) |  |  |  |  |
| Yes | 118 | 76 (71.0%) | 42 (71.2%) | 0.98 |
| No | 48 | 31 (29.0%) | 17 (28.8%) |  |
| BMI, n (%) |  |  |  |  |
| <18 | 3 | 3 (2.83%) | 0 (0.00%) | 0.25 |
| 18-25 | 100 | 59 (55.7%) | 41 (69.5%) |  |
| 25-30 | 41 | 29 (27.4%) | 12 (20.3%) |  |
| >30 | 21 | 15 (14.2%) | 6 (10.2%) |  |
| Smoking, n (%) |  |  |  |  |
| Yes | 16 | 14 (87.5%) | 2 (12.5%) | **0.045** |
| No | 151 | 94 (62.3%) | 57 (37.7%) |  |
| Alcohol, n (%) |  |  |  |  |
| 0 | 82 | 50 (46.7%) | 32 (54.2%) | 0.46 |
| 1-4 | 77 | 44 (41.1%) | 23 (39.0%) |  |
| >5 | 17 | 13 (12.2%) | 4 (6.78%) |  |
| HAD A, n (%) |  |  |  |  |
| <8 | 49 | 23 (45.1%) | 26 (44.1%) | 0.25 |
| 08-10 | 30 | 17 (33.3%) | 13 (22.0%) |  |
| >11 | 31 | 11 (21.6%) | 20 (33.9%) |  |
| HAD D, n (%) |  |  |  |  |
| <8 | 76 | 36 (70.6%) | 40 (67.8%) | 0.29 |
| 08-10 | 25 | 13 (25.5%) | 12 (20.3%) |  |
| >11 | 9 | 2 (3.92%) | 7 (11.9%) |  |

# Fig 2. Acceptability – Score of the Karasek model items, as a quantitative variable, using the Visual Analog Scale (VAS) and the Job-Demand-Control Support (JDCS) questionnaire of Karasek

*In the box plot (VAS in blue and JDCS in yellow), the lower and upper sides of the box are the lower and upper quartiles (Q1 and Q3). The box covers the interquartile interval (IQR), where 50% of the data is found. The horizontal line usually splits the box in two and is the median.*

# Table 1. Acceptability – Descriptive analysis of main outcome

| **Variables (test)** | **Sample size**  **N** | **Mean ± SD** | **Median [interquartile range]** | **Skewness** | **Kurtosis** | **Normality**  ***P-value*** |
| --- | --- | --- | --- | --- | --- | --- |
| **VAS** |  |  |  |  |  |  |
| *VAS Job-demand* | 141 | **24.1 ± 4.73** | **69 [50 ; 79]** | **-0.856** | **3.363** | **<0.001** |
| *VAS Job-demand* <58 | 46 (%) | 36.7 ± 17.5 | 43.5 [23 ; 50] | -0.739 | 2.311 |  |
| *VAS Job-demand* ≥ 58 | 95 (%) | 77.4 ± 10.9 | 76 [69 ; 84] | 0.589 | 2.449 |  |
| *VAS Job-control* | 139 | **65.6 ± 21.4** | **69 [51 ; 80]** | **-0.590** | **2.807** | **<0.001** |
| *VAS Job-control* <71.5 | 78 (%) | 51.4 ± 17.1 | 55 [40 ; 67] | -0.768 | 2.377 |  |
| *VAS Job-control* ≥71.5 | 61 (%) | 83.7 ± 9.42 | 83 [75 ; 91] | 0.345 | 1.757 |  |
| *VAS Social Support* | 150 | **51.6 ± 24.5** | **50.5 [29 ; 71]** | **-0.129** | **1.983** | **0.002** |
| *VAS Social Support* <63.5 | 94 (%) | 36.5 ± 17.1 | 37.5 [23 ; 50] | -0.768 | 2.377 |  |
| *VAS Social Support* ≥ 63.5 | 56 (%) | 76.8 ± 9.57 | 75 [68 ; 83] | 0.345 | 1.757 |  |
| **JDCS of Karasek** |  |  |  |  |  |  |
| *Job-demand* | 143 | **24.1± 4.73** | **24 [21 ; 27]** | **-0.064** | **2.462** | 0.96 |
| *Job-demand* < 21 | 35 (%) | 17.9±2.06 | 19 [17 ; 20] | -0.984 | 3.353 |  |
| *Job-demand* ≥ 21 | 108 (%) | 26.1±3.44 | 26 [23 ; 28] | 0.401 | 2.331 |  |
| *Job-control* | 138 | **69.9 ± 11.2** | **70 [62 ; 76]** | **-0.052** | **2.767** | 0.88 |
| *Job-control* <71 | 77 (%) | 62.1±7.13 | 64 [58 ; 68] | -0.928 | 3.375 |  |
| *Job-control* ≥71 | 61(%) | 79.8±6.49 | 78 [74 ; 84] | 0.572 | 2.363 |  |
| *Social Support* | 142 | **23.3 ± 4.57** | **24 [21 ; 26]** | **-0.338** | **3.192** | 0.13 |
| *Social Support* <24 | 89 (%) | 20.7±3.35 | 21 [19 ; 24] | -1.149 | 4.070 |  |
| *Social Support* ≥ 24 | 53 (%) | 27.8±2.30 | 28 [26 ; 29] | 0.624 | 2.263 |  |

# S1 Fig. Internal consistency and content validity – Correlation between i) the Visual Analog Scale (VAS); ii) between the VAS and the Job-Demand-Control Support (JDCS) questionnaire of Karasek; iii) Principal component analysis

*Factors that are located close together in the graph are well correlated. The PCA visually shows the proximity of VAS and JDCS for each sub-dimension.*

# Table 2. Internal validity – Cut-off determination for visual analog scales (VAS) and concordance with dimensions from the Job-Demand-Control Support (JDCS) questionnaire of Karasek

TP: True positive*; TN: True negative; FP: False positive; FN: False negative; Se: Sensibility (TP/(TP+FN)); Sp: Specificity (TN/(TN+FP); PPV: Positive predictive value (TP/(TP+FP); NPV: Negative predictive value (TN/(TN+FN); k: Kappa concordance coefficient

| **Variables** | **Cut-off** | **TP/TN** | **FP /FN** | **Se (%)** | **Sp (%)** | **PPV (%)** | **NPV (%)** | **%** | **k** |
| --- | --- | --- | --- | --- | --- | --- | --- | --- | --- |
| Job demand | 58 | 84/23 | 10/23 | 78.5  [69.5-85.9] | 69.7  [51.3-84.4] | 89.4  [81.3-94.8] | 50.0  [34.9-65.1] | 76.4% | 0.42 |
| Job control | 71.5 | 39/54 | 20/20 | 66.1  [52.6-77.9] | 73.0  [61.4-82.6] | 66.1  [52.6-77.9] | 73.0  [61.4-82.6] | 69.9% | 0.39 |
| Social support | 63.5 | 34/64 | 21/18 | 65.4  [50.9-78.0] | 75.3  [64.7-84.0] | 61.8  [47.7-74.6] | 78.0  [67.5-86.4] | 71.5% | 0.40 |
| Company support | 24.5 | 71/22 | 13/28 | 71.7  [61.8-80.3] | 62.9  [44.9-78.5] | 84.5  [75.0- 91.5] | 44.0  [30.0-58.7] | 69.4 % | 0.30 |
| Head support | 38.5 | 31/92 | 5/10 | 90.2  [82.7-95.2] | 86.1  [70.5-95.3] | 94.8  [88.4-98.3] | 75.6  [59.7-87.6] | 89.1% | 0.73 |
| Colleague support | 50.5 | 107/6 | 0/26 | 80.5  [72.7-86.8] | 100  [54.1-100] | 100.0  [96.6-100] | 18.8  [7.2-36.4] | 81.3% | 0.26 |

| **Variables** | **Cut-off** | **TP/TN** | **FP /FN** | **Se (%)** | **Sp (%)** | **PPV (%)** | **NPV (%)** | **%** | **k** |
| --- | --- | --- | --- | --- | --- | --- | --- | --- | --- |
| Job demand | 60 | 83/23 | 10/24 | 77.6  [68.56-85.1] | 69.7  [51.3-84.4] | 89.2  [81.1-94.7] | 49.0  [34.1-64.0] | 75.7% | 0.41 |
| Job control | 70 | 39/47 | 27/20 | 66.1  [52.6-77.9] | 63.5  [51.5-74.4] | 59.1  [46.3-71.0] | 70.1  [57.7-80.7] | 64.7% | 0.29 |
| Social support | 60 | 37/56 | 29/15 | 71.2  [56.9-82.9] | 65.9  [54.8-75.8] | 56.1  [43.3-68.3] | 78.9  [67.6-87.7] | 67.9% | 0.35 |

# S2 Table. Internal validity – Sensitivity analysis on rounded cut-off for visual analog scales (VAS) and concordance with dimensions from the Job-Demand-Control Support (JDCS) questionnaire of Karasek

TP: True positive*; TN: True negative; FP: False positive; FN: False negative; Se: Sensibility (TP/(TP+FN)); Sp: Specificity (TN/(TN+FP); PPV: Positive predictive value (TP/(TP+FP); NPV: Negative predictive value (TN/(TN+FN); k: Kappa concordance coefficient

# S2 Fig. Internal validity – Threshold determination for occupational stress using the visual analog scale (VAS) of job demand, job control and social support (new tool), in reference to the JDCS questionnaire of Karasek (gold standard) – Receptive operator characteristics (ROC) curve; and concordance between the VAS and the JDCS quadrants


**
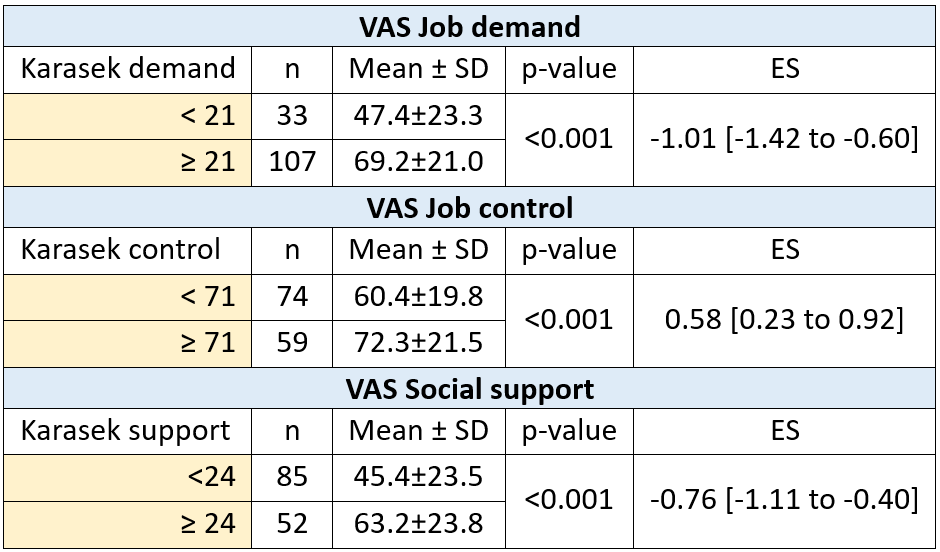
**

# Fig 5. Test-retest reproducibility – Main dimensions agreement between test and retest measures for the visual analog scale (VAS) (new tool), and for the JDCS score (gold standard) – Bland et Altman plots

*The horizontal axis represents the average score between test and retest for each dimension (VAS or JDCS). The vertical axis represents the difference between test and retest scores. The solid orange line shows the observed mean difference (average agreement), and the dashed lines indicate the 95% limits of agreement. The line at y = 0 represents perfect agreement between the two measurements.*

# S3 Fig. Test-retest reproducibility – Support sub-dimensions agreement between test and retest measures for the visual analog scale (VAS) (new tool), and for the JDCS score (gold standard) – Bland et Altman plots

*The horizontal axis represents the average score between test and retest for each dimension (VAS or JDCS). The vertical axis represents the difference between test and retest scores. The solid orange line shows the observed mean difference (average agreement), and the dashed lines indicate the 95% limits of agreement. The line at y = 0 represents perfect agreement between the two measurements.*

# S3 Table. Test-retest reproducibility – Sensitivity analysis comparing test–retest reliability coefficients for participants who completed the retest within 7 days (n = 28) versus after 7 days (n = 27)

|  |  | **Global** | **<7 days (n=28)** | **≥7 days (n=27)** |
| --- | --- | --- | --- | --- |
| VAS | Job demand | 0.65 | 0.59 | 0.78 |
|  | Job control | 0.79 | 0.94 | 0.53 |
|  | Social support | 0.46 | 0.45 | 0.42 |
|  | Head support | 0.86 | 0.89 | 0.79 |
|  | Colleague support | 0.87 | 0.89 | 0.85 |
|  | Company support | 0.68 | 0.72 | 0.52 |
| JDCS | Job demand | 0.84 | 0.85 | 0.82 |
|  | Job control | 0.89 | 0.90 | 0.87 |
|  | Social support | 0.86 | 0.95 | 0.77 |
|  | Head support | 0.87 | 0.92 | 0.81 |
|  | Colleague support | 0.78 | 0.85 | 0.70 |

# S4 Table. External validity – Relation of VAS and JDCS main items as continuous variables with secondary outcomes and agreements between measurement tools

*Mean±SD : Mean±Standard deviation*

| **Variables** | **Job demand** | | **Job control** | | **Social support** | |
| --- | --- | --- | --- | --- | --- | --- |
|  | VAS | Karasek | VAS | Karasek | VAS | Karasek |
|  | Mean ± SD | | Mean ± SD | | Mean ± SD | |
| **Age CL** |  |  |  |  |  |  |
| ≤40 | 61.9±24.3 | 23.8±4.86 | 68.0±19.6 | 70.6±11.1 | 55.4±25.3 | 24.2±4.31 |
| >40 | 66.8±22.2 | 24.4±4.55 | 62.7±23.3 | 69.3±11.2 | 47.6±23.0 | 22.4±4.73 |
| *p-value* | 0.17 | 0.45 | 0.15 | 0.51 | **0.04** | **0.02** |
| *Agreement* | High | | High | | High | |
| **Gender** |  |  |  |  |  |  |
| Women | 65.4±23.2 | 24.3±4.55 | 64.3±21.6 | 69.2±10.6 | 51.5±25.4 | 23.2±4.55 |
| Men | 60.4±23.7 | 23.3±5.21 | 69.5±20.7 | 72.0±12.6 | 51.7±21.8 | 23.6±4.69 |
| *p-value* | 0.20 | 0.31 | 0.17 | 0.25 | 0.96 | 0.72 |
| *Agreement* | High | | High | | High | |
| **Marital status** |  |  |  |  |  |  |
| Couple | 64.9±23.7 | 24.2±4.68 | 65.5±20.7 | 70.0±11.4 | 51.1±25.2 | 23.6±4.49 |
| Single | 61.6±22.1 | 23.6±4.89 | 65.8±25.1 | 69.0±10.4 | 51.9±20.7 | 22.4±4.93 |
| *p-value* | 0.30 | 0.50 | 0.73 | 0.66 | 0.86 | 0.25 |
| Agreement | High | | High | | High | |
| **Children** |  |  |  |  |  |  |
| 0 | 66.9±22.2 | 24.1±4.47 | 67.4±21.2 | 71.7±12.1 | 58.7±24.7 | 23.8±4.56 |
| 1 | 60.2±27.1 | 23.3±5.10 | 66.8±19.5 | 66.8±8.36 | 48.3±23.7 | 22.5±4/28 |
| ≥2 | 63.8±22.8 | 24.7±5.08 | 61.7±22.8 | 69.0±10.9 | 44.4±22.1 | 22.9±4.93 |
| *p-value* | 0.52 | 0.51 | 0.59 | 0.09 | **0.006** | 0.38 |
| *Agreement* | High | | Moderate | | Low | |
| **Education level** |  |  |  |  |  |  |
| ≤Bac | 68.5±17.3 | 25.2±4.06 | 58.9±16.3 | 61.6±6.98 | 43.5±19.9 | 21.4±2.98 |
| BAC +2/3 | 61.4±24.9 | 23.9±5.19 | 59.5±22.4 | 65.8±9.30 | 47.7±24.9 | 22.5±4.98 |
| BAC +5 | 61.0±23.8 | 24.3±S4.40 | 65.2±19.9 | 71.4±11.0 | 52.9±23.6 | 23.8±3.99 |
| > Bac+5 | 74.1±19.4 | 23.4±4.77 | 80.0±17.8 | 79.2±9.32 | 61.3±25.3 | 24.8±4.91 |
| *p-value* | 0.059 | 0.68 | **<0.001** | **<0.001** | **0.049** | 0.055 |
| *Agreement* | Moderate | | High | | Moderate | |
| **Occupation** |  |  |  |  |  |  |
| Executive / Intellectual | 67.5±22.8 | 24.3±4.69 | 71.3±19.2 | 74.5±9.88 | 57.4±23.6 | 24.1±4.42 |
| Intermediary | 61.1±24.2 | 24.1±4.86 | 58.0±22.8 | 64.8±9.80 | 45.9±23.5 | 22.7±4.78 |
| Employee | 51.8±18.1 | 24.8±4.60 | 59.8±9.91 | 61.6±11.1 | 33.2±20.0 | 19.6±2.79 |
| Student | 64.4±21.8 | 20.7±3.35 | 76.3±17.3 | 75.4±13.0 | 57.0±29.4 | 23.4±3.55 |
| *p-value* | 0.14 | 0.28 | **0.002** | **<0.001** | **0.01** | 0.08 |
| *Agreement* | High | | High | | Moderate | |
| **Working hours** |  |  |  |  |  |  |
| <30h | 56.8±16.6 | 20.6±4.98 | 65.1±22.4 | 68.5±12.5 | 50.4±21.9 | 24.5±4.15 |
| 30—40h | 62.5±24.4 | 23.9±4.44 | 65.7±21.9 | 69.2±11.1 | 51.8±25.2 | 22.8±4.67 |
| >40h | 73.5±20.6 | 26.7±4.23 | 65.4±20.0 | 73.2±10.1 | 51.6±24.0 | 24.5±4.25 |
| *p-value* | **0.005** | **<0.001** | 0.99 | 0.23 | 0.99 | 0.12 |
| *Agreement* | High | | High | | High | |
| **Management function** |  |  |  |  |  |  |
| No | 59.3±23.8 | 23.2±4.54 | 63.9±22.0 | 67.9±11.1 | 49.8±24.4 | 23.2±4.61 |
| Yes | 75.7±18.0 | 26.3±4.52 | 69.6±19.9 | 74.5±9.79 | 54.8±23.7 | 23.5±4.59 |
| *p-value* | **<0.001** | **<0.001** | 0.14 | **0.001** | 0.27 | 0.70 |
| Agreement | High | | Low | | High | |
| **BMI** |  |  |  |  |  |  |
| <18 | 80.5±3.54 | 27.3±3.06 | 79.0±9.90 | 77.3±7.02 | 58.0±21.8 | 22.3±1.53 |
| 18-25 | 65.8±21.2 | 23.8±4.91 | 64.8±22.2 | 70.4±11.4 | 53.4±24.9 | 23.7±4.96 |
| 25-30 | 59.6±25.8 | 23.8±4.23 | 68.4±18.7 | 68.7±9.09 | 46.4±22.9 | 22.8±3.87 |
| ≥30 | 65.1±27.7 | 25.3±5.00 | 61.4±23.9 | 67.4±13.3 | 52.2±28.0 | 22.2±4.25 |
| *p-value* | 0.51 | 0.36 | 0.43 | 0.43 | 0.47 | 0.53 |
| *Agreement* | High | | High | | High | |
| **Smoking** |  |  |  |  |  |  |
| No | 63.6±24.0 | 23.9±4.77 | 66.5±20.8 | 70.4±11.0 | 52.8±24.2 | 23.4±4.60 |
| Yes | 71.0±10.7 | 25.8±3.95 | 53.5±26.9 | 63.6±12.1 | 39.5±25.0 | 22.1±4.25 |
| *p-value* | 0.49 | 0.16 | 0.13 | 0.09 | 0.05 | 0.34 |
| Agreement | High | | Moderate | | Moderate | |
| **Alcohol** (glass per week) |  |  |  |  |  |  |
| 0 | 68.0±22.4 | 24.5±4 ?63 | 64.8±22.8 | 70.4±11.7 | 51.3±23.7 | 23.0±4.80 |
| 1-4 | 59.5±25.1 | 23.5±4.95 | 68.6±19.0 | 69.8±10.3 | 51.2±25.9 | 23.8±4.59 |
| ≥5 | 64.6±17.6 | 24.3±4.27 | 56.0±22.6 | 67.5±12.4 | 54.8±23.4 | 22.8±3.19 |
| *p-value* | 0.16 | 0.48 | 0.28 | 0.70 | 0.91 | 0.54 |
| *Agreement* | High | | High | | High | |
| **HAD-A** |  |  |  |  |  |  |
| <8 | 61.6±25.3 | 22.9±4.85 | 74.4±17.4 | 74.3±10.4 | 62.1±23.0 | 23.9±4.89 |
| 8-11 | 64.1±22.6 | 24.2±4.52 | 57.2±20.3 | 67.3±12.0 | 49.9±22.9 | 24.2±4.14 |
| ≥11 | 67.9±24.5 | 26.3±3.82 | 59.9±22.5 | 66.3±11.2 | 46.8±27.3 | 21.1±4.95 |
| *p-value* | 0.43 | **0.006** | **0.002** | **0.004** | **0.01** | **0.02** |
| *Agreement* | Low | | High | | High | |
| **HAD-D** |  |  |  |  |  |  |
| <8 | 64.2±24.0 | 24.4±4.65 | 69.5±20.3 | 73.3±10.9 | 60.5±23.7 | 24.3±4.55 |
| 8-11 | 66.6±22.9 | 26.4±4.43 | 60.6±17.3 | 64.1±8.79 | 42.5±22.0 | 22.0±3.74 |
| ≥11 | 56.6±30.5 | 25.1±3.68 | 45.7±25.2 | 57.0±9.74 | 32.6±22.9 | 16.9±5.38 |
| *p-value* | 0.75 | **0.02** | **0.004** | **<0.001** | **<0.001** | **<0.001** |
| *Agreement* | Low | | High | | High | |

| **p-value** | **color** |
| --- | --- |
| Non-significant (≥0.10) |  |
| Tendency (>0.05 to 0.10) |  |
| Significant (<0.05) |  |
| **Agreement** |  |
| Low (2 categories of differences) |  |
| Moderate (1category of differences) |  |
| High (same category) |  |

# S5 Table. External validity – Relations between VAS and JDCS main items as categorical variables with secondary outcomes variables and agreements between measurement tools

|  | **Job demand** | | | | **Job control** | | | | **Social support** | | | |
| --- | --- | --- | --- | --- | --- | --- | --- | --- | --- | --- | --- | --- |
| **Variables** | VAS n(%) | | Karasek n(%) | | VAS n(%) | | Karasek n(%) | | VAS n(%) | | Karasek n(%) | |
|  | <58 | ≥58 | <20 | ≥20 | <71.5 | ≥71.5 | <71 | ≥71 | <63.5 | ≥63.5 | <24 | ≥24 |
| **Age CL** |  |  |  |  |  |  |  |  |  |  |  |  |
| ≤40 | 26(58) | 49(52) | 19(56) | 55(51) | 38(49) | 36(59) | 37(49) | 36(59) | 41(44) | 38(68) | 38(43) | 35(66) |
| >40 | 19(42) | 46(48) | 15(44) | 53(49) | 39(51) | 25(41) | 39(51) | 25(41) | 52(56) | 18(32) | 50(57) | 18(34) |
| *Cramér's V* | 0.06 | | 0.04 | | 0.10 | | 0.10 | | 0.23 | | 0.22 | |
| *Agreement* | High | | | | High | | | | High | | | |
| **Gender** |  |  |  |  |  |  |  |  |  |  |  |  |
| Women | 33(72) | 73(77) | 22(63) | 85(79) | 64(82) | 41(67) | 62(81) | 42(69) | 70(74) | 42(75) | 67(75) | 39(74) |
| Men | 13(28) | 22(23) | 13(37) | 23(21) | 14(18) | 20(33) | 15(19) | 19(31) | 24(26) | 14(25) | 22(25) | 14(26) |
| *Cramér's V* | 0.06 | | 0.16 | | 0.17 | | 0.14 | | 0.01 | | 0.02 | |
| *Agreement* | High | | | | High | | | | High | | | |
| **Marital status** |  |  |  |  |  |  |  |  |  |  |  |  |
| Couple | 34(76) | 76(82) | 25(76) | 86(80) | 62(82) | 47(77) | 59(77) | 48(81) | 73(78) | 45(82) | 67(77) | 43(83) |
| Single | 11(24) | 17(18) | 8(24) | 21(20) | 14(18) | 14(23) | 18(23) | 11(19) | 20(22) | 10(12) | 20(23) | 9(17) |
| Cramér's V | 0.07 | | 0.05 | | 0.06 | | 0.06 | | 0.04 | | 0.07 | |
| Agreement | High | | | | High | | | | High | | | |
| **Children** |  |  |  |  |  |  |  |  |  |  |  |  |
| 0 | 18(45) | 50(53) | 16(47) | 53(51) | 39(51) | 29(51) | 30(40) | 36(61) | 35(39) | 36(64) | 40(47) | 29(57) |
| 1 | 11(26) | 14(15) | 9(26.5) | 19(18) | 15(19) | 10(18) | 22(30) | 5(8) | 19(21) | 10(18) | 20(23) | 7(14) |
| ≥2 | 12(29) | 30(32) | 9(26.5) | 32(31) | 23(30) | 18(31) | 22(30) | 18(31) | 35(40) | 10(18) | 26(30) | 15(29) |
| *Cramér's V* | 0.14 | | 0.09 | | 0.03 | | 0.28 | | 0.26 | | 0.13 | |
| *Agreement* | High | | | | Low | | | | Moderate | | | |
| **Education level** |  |  |  |  |  |  |  |  |  |  |  |  |
| ≤Bac | 2(4) | 10(11) | 2(6) | 12(11) | 10(23) | 2(3) | 14(18) | 0(0) | 13(14) | 2(4) | 12(13) | 2(4) |
| BAC +2/3 | 22(48) | 30(32) | 14(40) | 39(36) | 34(44) | 16(26) | 36(47) | 15(24) | 38(40) | 18(32) | 34(38) | 19(36) |
| BAC +5 | 18(39) | 31(33) | 11(31) | 37(34) | 30(38) | 19(31) | 22(29) | 23(38) | 30(32) | 21(38) | 30(34) | 17(32) |
| > Bac+5 | 4(9) | 24(25) | 8(23) | 20(19) | 4(5) | 24(39) | 5(6) | 23(38) | 13(14) | 15(27) | 13(15) | 15(28) |
| *Cramér's V* | 0.24 | | 0.09 | | 0.44 | | 0.49 | | 0.23 | | 0.21 | |
| *Agreement* | Moderate | | | | High | | | | High | | | |
| **Occupation** |  |  |  |  |  |  |  |  |  |  |  |  |
| Executive / Intellectual | 19(41) | 51(54) | 16(46) | 53(49) | 28(36) | 41(67) | 22(29) | 44(72) | 36(38) | 36(64) | 38(43) | 31(58) |
| Intermediary | 22(48) | 37(39) | 14(40) | 48(44) | 43(55) | 15(24) | 48(62) | 12(20) | 49(52) | 16(29) | 41(46) | 20(38) |
| Employee | 3(7) | 2(2) | 1(3) | 4(4) | 4(5) | 1(2) | 4(5) | 1(2) | 5(5) | 0(0) | 5(6) | 0(0) |
| Student | 2(4) | 5(5) | 4(11) | 3(3) | 3(4) | 4(7) | 3(4) | 4(7) | 4(4) | 4(7) | 5(6) | 2(4) |
| *Cramér's V* | 0.15 | | 0.17 | | 0.34 | | 0.46 | | 0.29 | | 0.20 | |
| *Agreement* | High | | | | High | | | | High | | | |
| **Working hours** |  |  |  |  |  |  |  |  |  |  |  |  |
| <30h | 8(17) | 8(8) | 8(23) | 8(7) | 9(12) | 7(11) | 11(14) | 5(8) | 10(11) | 8(14) | 8(9) | 8(15) |
| 30—40h | 33(72) | 63(66) | 25(71) | 73(68) | 53(68) | 41(67) | 58(75) | 37(61) | 65(69) | 36(64) | 66(74) | 32(60) |
| >40h | 5(11) | 24(25) | 2(6) | 27(25) | 16(21) | 13(21) | 8(10) | 19(31) | 19(20) | 12(21) | 15(17) | 13(25) |
| *Cramér's V* | 0.20 | | 0.27 | | 0.01 | | 0.26 | | 0.06 | | 0.15 | |
| *Agreement* | High | | | | Low | | | | High | | | |
| **Management fonction** |  |  |  |  |  |  |  |  |  |  |  |  |
| No | 41(89) | 58(62) | 29(85) | 72(67) | 59(77) | 38(62) | 63(82) | 35(58) | 68(72) | 38(69) | 60(68) | 40(75) |
| Yes | 5(11) | 36(38) | 5(15) | 36(33) | 18(23) | 23(38) | 14(18) | 25(42) | 26(28) | 17(31) | 28(32) | 13(25) |
| Cramér's V | 0.28 | | 0.18 | | 0.16 | | 0.26 | | 0.04 | | -0.08 | |
| Agreement | Moderate | | | | High | | | | High | | | |
| **BMI** |  |  |  |  |  |  |  |  |  |  |  |  |
| <18 | 0(0) | 2(2) | 0(0) | 3(3) | 0(0) | 2(3) | 1(1) | 2(3) | 1(1) | 2(4) | 3(3) | 0(0) |
| 18-25 | 26(58) | 57(60) | 23(66) | 61(57) | 45(58) | 36(60) | 42(55) | 40(67) | 54(58) | 37(66) | 45(51) | 39(75) |
| 25-30 | 15(33) | 21(22) | 8(23) | 28(26) | 18(23) | 18(30) | 25(32) | 9(15) | 27(29) | 10(18) | 28(31) | 8(15) |
| ≥30 | 4(9) | 15(16) | 4(11) | 15(14) | 15(19) | 4(7) | 9(12) | 9(15) | 11(12) | 7(12) | 13(15) | 5(10) |
| *Cramér's V* | 0.16 | | 0.11 | | 0.23 | | 0.21 | | 0.15 | | 0.25 | |
| *Agreement* | High | | | | High | | | | Moderate | | | |
| **Smoking** |  |  |  |  |  |  |  |  |  |  |  |  |
| No | 45(98) | 85(90) | 35(100) | 97(90) | 71(91) | 58(95) | 69(90) | 58(95) | 82(87) | 54(96) | 81(91) | 50(94) |
| Yes | 1(2) | 10(10) | 0(0) | 11(10) | 7(9) | 3(5) | 8(10) | 3(5) | 12(13) | 2(4) | 8(9) | 3(6) |
| Cramér's V | 0.15 | | 0.16 | | 0.08 | | 0.10 | | -0.15 | | -0.06 | |
| Agreement | High | | | | High | | | | High | | | |
| **Alcohol** (glass per week) |  |  |  |  |  |  |  |  |  |  |  |  |
| 0 | 19(41) | 50(53) | 15(43) | 56(52) | 38(49) | 30(49) | 39(51) | 30(49) | 48(51) | 25(45) | 45(31) | 26(49) |
| 1-4 | 24(52) | 34(36) | 17(49) | 41(38) | 32(41) | 26(43) | 30(39) | 27(44) | 37(39) | 25(45) | 34(38) | 23(43) |
| ≥5 | 3(7) | 11(11) | 3(9) | 11(10) | 8(10) | 5(8) | 8(10) | 5(7) | 9(10) | 6(10) | 10(11) | 4(8) |
| *Cramér's V* | 0.16 | | 0.09 | | 0.04 | | 0.08 | | 0.06 | | 0.07 | |
| *Agreement* | High | | | | High | | | | High | | | |
| **HAD-A** |  |  |  |  |  |  |  |  |  |  |  |  |
| <8 | 18(53) | 29(40) | 15(58) | 32(40) | 17(28) | 30(65) | 18(30) | 27(60) | 19(32) | 28(62) | 25(38) | 22(54) |
| 8-11 | 10(29) | 20(27) | 8(31) | 22(27) | 23(38) | 7(15) | 20(33) | 10(22) | 18(31) | 10(22) | 17(26) | 13(32) |
| ≥11 | 6(18) | 24(33) | 3(11) | 27(33) | 21(34) | 9(20) | 22(37) | 8(18) | 22(37) | 7(16) | 24(36) | 6(14) |
| *Cramér's V* | 0.16 | | 0.21 | | 0.38 | | 0.30 | | 0.31 | | 0.24 | |
| *Agreement* | High | | | | High | | | | High | | | |
| **HAD-D** |  |  |  |  |  |  |  |  |  |  |  |  |
| <8 | 24(71) | 50(68) | 22(85) | 53(65) | 37(61) | 37(80) | 34(57) | 40(89) | 35(59) | 39(87) | 40(61) | 35(85) |
| 8-11 | 8(24) | 16(22) | 3(12) | 21(26) | 16(26) | 8(17) | 18(30) | 5(11) | 17(29) | 5(11) | 18(27) | 6(15) |
| ≥11 | 2(6) | 7(10) | 1(4) | 7(9) | 8(13) | 1(2) | 8(13) | 0(0) | 7(12) | 1(2) | 8(12) | 0(0) |
| *Cramér's V* | 0.06 | | 0.18 | | 0.24 | | 0.37 | | 0.30 | | 0.29 | |
| *Agreement* | Moderate | | | | Moderate | | | | High | | | |

| **Cramér's V** | **color** |
| --- | --- |
| Very low (<0.10) |  |
| Low (≥0.10 to <0.20) |  |
| Moderate (≥0.20 to <0.30) |  |
| High (≥0.30) |  |
| **Agreement** |  |
| Low (≥0.2 points difference) |  |
| Moderate (0.1 to 0.2 points difference) |  |
| High (≤0.1 points difference) |  |

# Fig 6. External validity – Relations between VAS and JDCS main items as categorical variables with secondary outcomes variables and agreements between measurement tools illustrated using a polar plot

*The prevalence of high demand, low control and low support was compared between groups using a Chi² test. To quantify the strength of the association between secondary outcomes and each dimension, Cramer’s V was calculated. Agreement was considered low for ≥0.2 points difference, moderate for 0.1 to 0.2 points difference, and high for ≤0.1* *points difference.*

# S4 Fig. External validity – Relations between VAS and JDCS main items as continuous variables with secondary outcomes variables and agreements between measurement tools illustrated using a forest plot

*The effect of each variable is represented by a dot on a horizontal line. The dots represent the effect (coefficient) for each variable, and the line around the dots their 95% confidence interval (95CI). The vertical line represents the null estimate (with a value of 0). Coefficients with horizontal lines that do not cross the null vertical line are significant. Significant variables with a coefficient <0 are protective factors and those with a coefficient >0 are risk factors. REF: Reference i.e. the reference for group comparisons.*

# S6 Table. External validity – Relations between VAS and JDCS sub-dimensions items according to cut-offs with other variables and agreements between measurement tools

| **Variables** | **Hierarchy support** | | | | | | **Colleague support** | | | |
| --- | --- | --- | --- | --- | --- | --- | --- | --- | --- | --- |
|  | VAS from direct supervisor n(%) | | Karasek n(%) | | VAS from company n(%) | | VAS n(%) | | Karasek n(%) | |
|  | <38.5 | ≥38.5 | ≤8 | >8 | <24.5 | ≥24.5 | <50.5 | ≥50.5 | ≤8 | >8 |
| **Age CL** |  |  |  |  |  |  |  |  |  |  |
| ≤40 | 18(44) | 56(57) | 14(39) | 59(56) | 25(49) | 47(56) | 16(48) | 59(55) | 4(57) | 69(51) |
| >40 | 23(56) | 42(43) | 22(61) | 46(44) | 26(51) | 37(44) | 17(51) | 48(45) | 3(43) | 65(49) |
| *Cramér's V* | 0.12 | | 0.15 | | 0.07 | | 0.06 | | 0.03 | |
| *Agreement* | High | | | |  | | High | | | |
|  |  | | High | | | |  |  |  |  |
| **Gender** |  |  |  |  |  |  |  |  |  |  |
| Women | 35(85) | 70(71) | 28(78) | 78(74) | 39(76) | 63(74) | 23(70) | 83(77) | 5(71) | 101() |
| Men | 6(15) | 29(29) | 8(22) | 28(26) | 12(24) | 22(26) | 10(30) | 25(23) | 2(29) | 34(25) |
| *Cramér's V* | 0.15 | | 0.04 | | 0.03 | | 0.07 | | 0.02 | |
| *Agreement* | Moderate | | | |  | | High | | | |
|  |  | | High | | | |  |  |  |  |
| **Marital status** |  |  |  |  |  |  |  |  |  |  |
| Couple | 29(73) | 80(82) | 24(69) | 86(83) | 38(75) | 70(83) | 25(78) | 85(80) | 3(43) | 107(81) |
| Single | 11(27) | 18(18) | 11(31) | 18(17) | 13(25) | 14(17) | 7(22) | 21(20) | 5(57) | 25(19) |
| Cramér's V | -0.10 | | -0.15 | | -0.12 | | 0.02 | | 0.21 | |
| Agreement | High | | | |  | | Moderate | | | |
|  |  | | High | | | |  |  |  |  |
| **Children** |  |  |  |  |  |  |  |  |  |  |
| 0 | 19(48) | 49(51) | 19(54) | 50(49) | 20(42) | 46(55) | 11(35) | 58(55) | 3(43) | 66(51) |
| 1 | 10(26) | 16(17) | 8(23) | 19(19) | 12(25) | 13(16) | 7(23) | 18(17) | 1(14) | 25(20) |
| ≥2 | 10(26) | 31(32) | 8(23) | 33(32) | 16(33) | 24(29) | 13(42) | 29(28) | 3(43) | 38(29) |
| *Cramér's V* | 0.11 | | 0.09 | | 0.14 | | 0.17 | | 0.07 | |
| *Agreement* | High | | | |  | | Moderate | | | |
|  |  |  | High | | | |  |  |  |  |
| **Education level** |  |  |  |  |  |  |  |  |  |  |
| ≤Bac | 4(10) | 8(8) | 3(8) | 11(10) | 8(16) | 5(6) | 4(12) | 9(8) | 0(0) | 14(10) |
| BAC +2/3 | 18(43) | 33(33) | 16(44) | 37(35) | 25(49) | 24(28) | 16(48) | 35(32) | 3(43) | 50(37) |
| BAC +5 | 12(30) | 37(37) | 12(33) | 35(33) | 12(24) | 34(40) | 11(33) | 38(35) | 4(43) | 44(33) |
| > Bac+5 | 7(17) | 21(21) | 5(14) | 23(22) | 6(11) | 22(26) | 2(6) | 26(24) | 1(14) | 27(20) |
| *Cramér's V* | 0.11 | | 0.11 | | 0.31 | | 0.21 | | 0.09 | |
| *Agreement* | High | | | |  | | Moderate | | | |
|  |  | | Low | | | |  |  |  |  |
| **Occupation** |  |  |  |  |  |  |  |  |  |  |
| Executive / Intellectual | 16(39) | 54(55) | 15(42) | 54(51) | 18(35) | 49(58) | 13(40) | 57(53) | 3(43) | 66(49) |
| Intermediary | 21(51) | 37(37) | 18(50) | 43(41) | 30(59) | 28(33) | 16(48) | 43(40) | 3(43) | 58(43) |
| Employee | 2(5) | 3(3) | 2(6) | 3(3) | 2(4) | 3(3) | 3(9) | 2(2) | 0(0) | 5(4) |
| Student | 2(5) | 5(5) | 1(3) | 6(5) | 1(2) | 5(6) | 1(3) | 6(5) | 1(14) | 6(4) |
| *Cramér's V* | 0.15 | | 0.12 | | 0.26 | | 0.20 | | 0.11 | |
| *Agreement* | High | | | |  | | High | | | |
|  |  | | Moderate | | | |  |  |  |  |
| **Working hours** |  |  |  |  |  |  |  |  |  |  |
| <30h | 2(5) | 15(15) | 3(8) | 13(12) | 4(8) | 12(14) | 3(9) | 14(13) | 1(14) | 15(11) |
| 30—40h | 32(78) | 62(63) | 29(81) | 69(65) | 41(80) | 50(59) | 24(72) | 71(66) | 6(86) | 92(68) |
| >40h | 7(17) | 22(22) | 4(11) | 24(23) | 6(12) | 23(27) | 6(18) | 23(21) | 0(0) | 28(21) |
| *Cramér's V* | 0.17 | | 0.15 | | 0.22 | | 0.07 | | 0.11 | |
| *Agreement* | High | | | |  | | High | | | |
|  |  | | High | | | |  |  |  |  |
| **Management fonction** |  |  |  |  |  |  |  |  |  |  |
| No | 30(73) | 69(70) | 28(78) | 72(69) | 42(82) | 53(62) | 24(72) | 75(70) | 5(71) | 95(70) |
| Yes | 11(27) | 29(30) | 8(22) | 33(31) | 9(18) | 32(38) | 8(27) | 32(30) | 2(29) | 39(29) |
| Cramér's V | 0.03 | | 0.09 | | 0.21 | | 0.03 | | 0.00 | |
| Agreement | High | | | |  | | High | | | |
|  |  | | Moderate | | | |  |  |  |  |
| **BMI** |  |  |  |  |  |  |  |  |  |  |
| <18 | 2(5) | 0(0) | 2(6) | 1(1) | 0(0) | 2(2) | 0(0) | 2(2) | 0(0) | 3(2) |
| 18-25 | 22(54) | 61(62) | 19(53) | 65(62) | 26(51) | 54(64) | 18(55) | 65(61) | 6(86) | 78(58) |
| 25-30 | 12(29) | 23(23) | 9(25) | 27(26) | 19(37) | 17(20) | 9(27) | 27(25) | 0(0) | 36(27) |
| ≥30 | 5(12) | 14(14) | 6(16) | 12(11) | 6(12) | 11(13) | 6(18) | 13(12) | 1(14) | 17(13) |
| *Cramér's V* | 0.20 | | 0.16 | | 0.21 | | 0.10 | | 0.14 | |
| *Agreement* | High | | | |  | | High | | | |
|  |  | | High | | | |  |  |  |  |
| **Smoking** |  |  |  |  |  |  |  |  |  |  |
| No | 39(95) | 91(92) | 34(94) | 97(92) | 46(90) | 80(94) | 29(88) | 102(94) | 6(86) | 125(93) |
| Yes | 2(5) | 8(8) | 2(6) | 9(5) | 5(10) | 5(6) | 4(12) | 6(6) | 1(14) | 10(7) |
| Cramér's V | 0.06 | | 0.05 | | 0.07 | | 0.11 | | 0.06 | |
| Agreement | High | | | |  | | High | | | |
|  |  | | High | | | |  |  |  |  |
| **Alcohol** (glass per week) |  |  |  |  |  |  |  |  |  |  |
| 0 | 20(49) | 49(49) | 16(44) | 55(52) | 25(49) | 42(49) | 16(48) | 54(50) | 7(100) | 64(47) |
| 1-4 | 18(44) | 40(40) | 16(44) | 41(39) | 21(41) | 36(42) | 14(42) | 44(41) | 0(0) | 57(42) |
| ≥5 | 3(7) | 10(10) | 4(12) | 10(9) | 5(10) | 7(8) | 3(9) | 10(9) | 0(0) | 14(11) |
| *Cramér's V* | 0.05 | | 0.07 | | 0.03 | | 0.02 | | 0.23 | |
| *Agreement* | High | | | |  | | Low | | | |
|  |  | | High | | | |  |  |  |  |
| **HAD-A** |  |  |  |  |  |  |  |  |  |  |
| <8 | 10(29) | 38(52) | 10(33) | 37(48) | 13(36) | 34(49) | 8(31) | 40(49) | 1(17) | 46(45) |
| 8-11 | 8(24) | 21(29) | 7(23) | 23(30) | 12(32) | 18(26) | 5(19) | 25(30) | 2(33) | 28(28) |
| ≥11 | 16(47) | 14(19) | 13(43) | 17(22) | 12(32) | 17(25) | 13(50) | 17(21) | 3(50) | 27(27) |
| *Cramér's V* | 0.30 | | 0.21 | | 0.14 | | 0.28 | | 0.14 | |
| *Agreement* | High | | | |  | | Moderate | | | |
|  |  | | High | | | |  |  |  |  |
| **HAD-D** |  |  |  |  |  |  |  |  |  |  |
| <8 | 17(50) | 57(78) | 14(47) | 61(79) | 17(46) | 56(81) | 13(50) | 62(75) | 2(33) | 73(72) |
| 8-11 | 11(32) | 13(18) | 10(33) | 14(18) | 13(35) | 11(16) | 7(27) | 17(21) | 1(17) | 23(23) |
| ≥11 | 6(18) | 3(4) | 6(20) | 2(3) | 7(19) | 2(3) | 6(23) | 3(4) | 3(50) | 5(5) |
| *Cramér's V* | 0.30 | | 0.37 | | 0.38 | | 0.32 | | 0.40 | |
| *Agreement* | High | | | |  | | High | | | |
|  |  | | High | | | |  |  |  |  |

| **Cramér's V** | **color** |
| --- | --- |
| Very low (<0.10) |  |
| Low (≥0.10 to <0.20) |  |
| Moderate (≥0.20 to <0.30) |  |
| High (≥0.30) |  |
| **Agreement** |  |
| Low (≥0.2 points difference) |  |
| Moderate (0.1 to 0.2 points difference) |  |
| High (≤0.1 points difference) |  |

# S7 Table. External validity – Relations between VAS and JDCS treated as quadrants with secondary outcomes variables and agreements between measurement tools

| **Variables** | **VAS quadrants** | | | | **VAS Karasek** | | | |
| --- | --- | --- | --- | --- | --- | --- | --- | --- |
|  | Jobstrain | Passive | Active | Low strain | Jobstrain | Passive | Active | Low strain |
| **Age CL** |  |  |  |  |  |  |  |  |
| ≤40 | 24(47) | 14(54) | 25(59) | 11(61) | 29(49) | 8(47) | 25(56) | 11(69) |
| >40 | 27(53) | 12(46) | 17(41) | 7(39) | 30(51) | 9(53) | 20(44) | 5(31) |
| *Cramér's V* | 0.12 | | | | 0.13 | | | |
| *Agreement* | High | | | | | | | |
| **Gender** |  |  |  |  |  |  |  |  |
| Women | 44(86) | 20(74) | 28(67) | 12(67) | 51(86) | 11(61) | 32(71) | 10(63) |
| Men | 7(14) | 7(26) | 14(33) | 6(33) | 8(14) | 7(39) | 13(29) | 6(37) |
| *Cramér's V* | 0.20 | | | | 0.24 | | | |
| *Agreement* | High | | | | | | | |
| **Marital status** |  |  |  |  |  |  |  |  |
| Couple | 41(82) | 21(81) | 34(81) | 13(72) | 46(78) | 13(72) | 36(82) | 12(80) |
| Single | 9(18) | 5(19) | 5(19) | 5(28) | 13(22) | 5(28) | 8(18) | 8(20) |
| Cramér's V | 0.09 | | | | 0.07 | | | |
| Agreement | High | | | | | | | |
| **Children** |  |  |  |  |  |  |  |  |
| 0 | 26(52) | 13(48) | 23(55) | 6(42) | 24(42) | 6(35) | 27(63) | 9(56) |
| 1 | 8(16) | 7(26) | 5(12) | 4(29) | 13(23) | 9(53) | 5(12) | 0(0) |
| ≥2 | 16(32) | 7(26) | 14(33) | 4(29) | 20(35) | 2(12) | 11(25) | 7(44) |
| *Cramér's V* | 0.11 | | | | 0.28 | | | |
| *Agreement* | Moderate | | | | | | | |
| **Education level** |  |  |  |  |  |  |  |  |
| ≤Bac | 9(18) | 1(4) | 1(2) | 1(6) | 12(20) | 2(11) | 0(0) | 0(0) |
| BAC +2/3 | 20(39) | 14(52) | 8(19) | 7(39) | 26(44) | 10(56) | 12(27) | 3(19) |
| BAC +5 | 19(37) | 11(40) | 12(29) | 7(39) | 18(31) | 4(22) | 16(36) | 7(44) |
| > Bac+5 | 3(6) | 1(4) | 51(50) | 3(16) | 3(5) | 2(11) | 17(37) | 6(37) |
| *Cramér's V* | 0.32 | | | | 0.29 | | | |
| *Agreement* | High | | | | | | | |
| **Occupation** |  |  |  |  |  |  |  |  |
| Executive / Intellectual | 19(37) | 9(33) | 32(76) | 9(50) | 16(27) | 6(33) | 34(76) | 10(63) |
| Intermediary | 28(55) | 15(56) | 7(17) | 7(38) | 38(64) | 10(56) | 9(20) | 3(19) |
| Employee | 2(4) | 2(7) | 0(0) | 1(6) | 3(5) | 1(6) | 1(2) | 0(0) |
| Student | 2(4) | 1(4) | 3(7) | 1(6) | 2(3) | 1(6) | 1(2) | 3(18) |
| *Cramér's V* | 0.23 | | | | 0.30 | | | |
| *Agreement* | High | | | | | | | |
| **Working hours** |  |  |  |  |  |  |  |  |
| <30h | 5(10) | 4(15) | 3(7) | 4(22) | 8(14) | 3(17) | 0(0) | 5(31) |
| 30—40h | 34(67) | 19(70) | 27(64) | 13(72) | 43(72) | 15(83) | 28(62) | 9(56) |
| >40h | 12(23) | 4(15) | 12(29) | 1(6) | 8(14) | 0(0) | 17(38) | 2(13) |
| *Cramér's V* | 0.16 | | | | 0.30 | | | |
| *Agreement* | Moderate | | | | | | | |
| **Management fonction** |  |  |  |  |  |  |  |  |
| No | 34(68) | 25(93) | 22(52) | 15(83) | 46(78) | 17(94) | 24(53) | 11(73) |
| Yes | 16(32) | 2(7) | 20(48) | 3(17) | 13(22) | 1(6) | 21(47) | 4(27) |
| Cramér's V | 0.32 | | | | 0.31 | | | |
| Agreement | High | | | | | | | |
| **BMI** |  |  |  |  |  |  |  |  |
| <18 | 0(0) | 0(0) | 2(5) | 0(0) | 1(2) | 0(0) | 2(5) | 0(0) |
| 18-25 | 28(55) | 17(63) | 27(64) | 8(47) | 35(54) | 10(56) | 28(64) | 12(74) |
| 25-30 | 12(24) | 6(22) | 9(21) | 9(53) | 19(32) | 6(33) | 7(16) | 2(13) |
| ≥30 | 11(22) | 4(15) | 4(10) | 0(0) | 7(12) | 2(11) | 7(16) | 2(13) |
| *Cramér's V* | 0.20 | | | | 0.14 | | | |
| *Agreement* | High | | | | | | | |
| **Smoking** |  |  |  |  |  |  |  |  |
| No | 45(88) | 26(96) | 39(93) | 18(100) | 51(86) | 18(100) | 42(93) | 16(100) |
| Yes | 6(12) | 1(4) | 3(7) | 0(0) | 8(14) | 0(0) | 3(7) | 0(0) |
| Cramér's V | 0.16 | | | | 0.20 | | | |
| Agreement | High | | | | | | | |
| **Alcohol** (glass per week) |  |  |  |  |  |  |  |  |
| 0 | 27(53) | 11(41) | 22(52) | 7(39) | 31(53) | 5(44) | 23(51) | 7(44) |
| 1-4 | 18(35) | 14(52) | 16(38) | 10(56) | 20(34) | 10(56) | 21(47) | 6(38) |
| ≥5 | 6(12) | 2(7) | 4(10) | 1(5) | 8(13) | 0(0) | 1(2) | 3(18) |
| *Cramér's V* | 0.12 | | | | 0.19 | | | |
| *Agreement* | High | | | | | | | |
| **HAD-A** |  |  |  |  |  |  |  |  |
| <8 | 9(23) | 8(38) | 20(61) | 10(77) | 11(24) | 7(50) | 20(58) | 7(64) |
| 8-11 | 14(35) | 9(43) | 6(18) | 1(8) | 15(33) | 5(36) | 7(21) | 3(27) |
| ≥11 | 17(42) | 4(19) | 7(21) | 7(15) | 20(43) | 2(14) | 7(21) | 1(9) |
| *Cramér's V* | 0.31 | | | | 0.27 | | | |
| *Agreement* | High | | | | | | | |
| **HAD-D** |  |  |  |  |  |  |  |  |
| <8 | 23(58) | 14(67) | 27(82) | 10(77) | 24(52) | 10(71) | 29(85) | 11(100) |
| 8-11 | 11(27) | 5(24) | 5(15) | 3(23) | 15(33) | 3(21) | 5(15) | 0(0) |
| ≥11 | 6(15) | 2(9) | 1(3) | 0(0) | 7(15) | 1(7) | 0(0) | 0(0) |
| *Cramér's V* | 0.18 | | | | 0.29 | | | |
| *Agreement* | High | | | | | | | |

| **Cramér's V** | **color** |
| --- | --- |
| Very low (<0.10) |  |
| Low (≥0.10 to <0.20) |  |
| Moderate (≥0.20 to <0.30) |  |
| High (≥0.30) |  |
| **Agreement** |  |
| Low (≥0.2 points difference) |  |
| Moderate (0.1 to 0.2 points difference) |  |
| High (≤0.1 points difference) |  |

# Table 4. External validity – Relation between VAS and JDCS (treated as Jobstrain and Isostrain) with secondary outcomes variables and agreements between measurement tools

| **Variables** | **Jobstrain** | | | | **Isostrain** | | | |
| --- | --- | --- | --- | --- | --- | --- | --- | --- |
|  | VAS n(%) | | Karasek n(%) | | VAS n(%) | | Karasek n(%) | |
|  | No jobstrain | Jobstrain | No jobstrain | Jobstrain | No isostrain | Isostrain | No isostrain | Isostrain |
| **Age CL** |  |  |  |  |  |  |  |  |
| ≤40 | 50(58) | 24(47) | 44(56) | 29(49) | 9(60) | 15(42) | 8(62) | 21(46) |
| >40 | 36(42) | 27(53) | 34(44) | 30(51) | 6(40) | 21(58) | 5(38) | 25(54) |
| *Cramér’s V* | 0.11 | | 0.07 | | 0.17 | | 0.13 | |
| *Agreement* | High | | | | High | | | |
| **Gender** |  |  |  |  |  |  |  |  |
| Women | 60(69) | 44(86) | 53(67) | 51(86) | 14(93) | 30(83) | 13(100) | 38(83) |
| Men | 27(31) | 7(14) | 26(33) | 8(14) | 1(7) | 6(17) | 0(0) | 8(17) |
| *Cramér’s V* | 0.19 | | 0.22 | | 0.13 | | 0.21 | |
| *Agreement* | High | | | | High | | | |
| **Marital status** |  |  |  |  |  |  |  |  |
| Couple | 68(79) | 41(82) | 61(79) | 46(78) | 12(86) | 29(81) | 10(77) | 36(78) |
| Single | 18(21) | 9(18) | 16(21) | 13(22) | 2(14) | 7(19) | 3(23) | 10(22) |
| *Cramér’s V* | 0.03 | | 0.02 | | 0.06 | | 0.01 | |
| Agreement | High | | | | High | | | |
| **Children** |  |  |  |  |  |  |  |  |
| 0 | 42(50) | 26(52) | 42(55) | 24(42) | 11(73) | 15(43) | 5(42) | 19(42) |
| 1 | 16(19) | 8(16) | 14(18) | 13(23) | 2(13) | 6(17) | 4(33) | 9(20) |
| ≥2 | 25(30) | 16(32) | 20(26) | 20(35) | 2(13) | 14(40) | 3(25) | 17(38) |
| *Cramér’s V* | 0.04 | | 0.13 | | 0.29 | | 0.14 | |
| *Agreement* | High | | | | Moderate | | | |
| **Education level** |  |  |  |  |  |  |  |  |
| ≤Bac | 3(3) | 9(18) | 2(2) | 12(20) | 2(13) | 7(19) | 2(15) | 10(22) |
| BAC +2/3 | 29(33) | 20(39) | 25(32) | 26(44) | 8(53) | 12(33) | 6(46) | 20(43) |
| BAC +5 | 30(34) | 19(37) | 27(34) | 18(31) | 5(33) | 14(39) | 4(31) | 14(30) |
| > Bac+5 | 25(29) | 3(6) | 25(32) | 3(5) | 0(0) | 3(8) | 1(8) | 2(4) |
| *Cramér’s V* | 0.34 | | 0.42 | | 0.23 | | 0.09 | |
| *Agreement* | High | | | | Moderate | | | |
| **Occupation** |  |  |  |  |  |  |  |  |
| Executive / Intellectual | 50(57) | 19(37) | 50(63) | 16(27) | 5(33) | 14(39) | 3(23) | 13(28) |
| Intermediary | 29(33) | 28(55) | 22(28) | 38(64) | 9(60) | 19(53) | 10(77) | 28(61) |
| Employee | 3(3) | 2(4) | 2(2) | 3(5) | 0(0) | 2(6) | 0(0) | 3(7) |
| Student | 5(6) | 2(4) | 5(6) | 2(3) | 1(7) | 1(3) | 0(0) | 2(4) |
| *Cramér’s V* | 0.22 | | 0.39 | | 0.17 | | 0.18 | |
| *Agreement* | Moderate | | | | High | | | |
| **Working hours** |  |  |  |  |  |  |  |  |
| <30h | 11(13) | 5(10) | 8(10) | 8(14) | 2(13) | 3(8) | 4(31) | 7(9) |
| 30—40h | 59(68) | 34(67) | 52(66) | 43(73) | 9(60) | 25(69) | 6(46) | 37(80) |
| >40h | 17(19) | 12(23) | 19(24) | 8(14) | 4(27) | 8(22) | 3(23) | 5(11) |
| *Cramér’s V* | 0.06 | | 0.13 | | 0.10 | | 0.33 | |
| *Agreement* | High | | | | Low | | | |
| **Management fonction** |  |  |  |  |  |  |  |  |
| No | 62(71) | 34(68) | 52(67) | 46(78) | 9(64) | 25(69) | 13(100) | 33(72) |
| Yes | 25(29) | 16(32) | 26(33) | 13(22) | 5(36) | 11(31) | 0(0) | 13(28) |
| *Cramér’s V* | 0.03 | | 0.12 | | 0.05 | | 0.28 | |
| Agreement | High | | | | Low | | | |
| **BMI** |  |  |  |  |  |  |  |  |
| <18 | 2(2) | 0(0) | 2(3) | 1(2) | 0(0) | 0(0) | 0(0) | 1(2) |
| 18-25 | 52(60) | 28(55) | 50(64) | 32(54) | 6(40) | 22(61) | 8(62) | 24(52) |
| 25-30 | 24(28) | 12(24) | 15(19) | 19(32) | 3(20) | 9(25) | 3(23) | 16(35) |
| ≥30 | 8(9) | 11(22) | 11(14) | 7(12) | 6(40) | 5(14) | 2(15) | 5(11) |
| *Cramér’s V* | 0.19 | | 0.15 | | 0.29 | | 0.13 | |
| *Agreement* | High | | | | Moderate | | | |
| **Smoking** |  |  |  |  |  |  |  |  |
| No | 83(95) | 45(88) | 76(96) | 51(86) | 13(87) | 32(89) | 11(85) | 40(87) |
| Yes | 4(5) | 6(12) | 7(4) | 8(14) | 2(13) | 4(11) | 2(15) | 6(13) |
| *Cramér’s V* | 0.13 | | 0.18 | | 0.03 | | 0.03 | |
| Agreement | High | | | | High | | | |
| **Alcohol** (glass per week) |  |  |  |  |  |  |  |  |
| 0 | 40(46) | 27(53) | 38(48) | 21(52) | 4(27) | 23(64) | 5(38) | 26(57) |
| 1-4 | 40(46) | 18(35) | 37(47) | 20(34) | 9(60) | 9(25) | 7(54) | 13(28) |
| ≥5 | 7(8) | 6(12) | 4(5) | 8(14) | 2(13) | 4(11) | 1(8) | 7(15) |
| *Cramér’s V* | 0.11 | | 0.18 | | 0.36 | | 0.23 | |
| *Agreement* | High | | | | Moderate | | | |
| **HAD-A** |  |  |  |  |  |  |  |  |
| <8 | 38(57) | 9(22) | 34(58) | 11(24) | 5(42) | 4(14) | 1(10) | 10(28) |
| 8-11 | 16(24) | 14(35) | 15(25) | 15(33) | 4(33) | 10(36) | 6(60) | 9(25) |
| ≥11 | 13(19) | 17(43) | 10(17) | 20(43) | 3(25) | 14(50) | 3(30) | 17(47) |
| *Cramér’s V* | 0.34 | | 0.36 | | 0.32 | | 0.31 | |
| *Agreement* | High | | | | High | | | |
| **HAD-D** |  |  |  |  |  |  |  |  |
| <8 | 51(76) | 23(58) | 50(85) | 24(52) | 9(75) | 14(50) | 5(50) | 19(53) |
| 8-11 | 13(19) | 11(27) | 8(13) | 15(33) | 2(17) | 9(32) | 5(50) | 10(28) |
| ≥11 | 9(4) | 6(15) | 1(2) | 7(15) | 1(8) | 5(18) | 0(0) | 7(19) |
| *Cramér’s V* | 0.22 | | 0.37 | | 0.23 | | 0.26 | |
| *Agreement* | Moderate | | | | High | | | |

| **Cramér's V** | **color** |
| --- | --- |
| Very low (<0.10) |  |
| Low (≥0.10 to <0.20) |  |
| Moderate (≥0.20 to <0.30) |  |
| High (≥0.30) |  |
| **Agreement** |  |
| Low (≥0.2 points difference) |  |
| Moderate (0.1 to 0.2 points difference) |  |
| High (≤0.1 points difference) |  |

# S5 Fig. External validity – Relation between VAS and JDCS (treated as Jobstrain and Isostrain) with secondary outcome variables illustrated using a polar plot

*The prevalence of Jobstrain and Isostrain was compared between groups using a Chi² test.*

# Fig 7. External validity – Relation between VAS and JDCS (treated as Jobstrain and Isostrain) with secondary outcome variables, and agreement between measurement tools illustrated using a forest plot

*The effect of each variable on the risk of Jobstrain / Isostrain is represented by a dot on a horizontal line. The dots represent the risk of Jobstrain or Isostrain (odds ratio) for each variable, and the line around the dots represent their 95% confidence interval (95CI). The vertical line represents the null estimate (with a value of 1). Odds ratio with horizontal lines that do not cross the vertical line are significant. Significant variables with an odds ratio <1 are protective factors and those with an odds ratio >1 are risk factors. REF: Reference i.e. the reference for group comparisons.*
