# Supplementary material for: Validation of visual analogue scales to assess occupational stress compared to the Karasek questionnaire: A cross sectional study
Source: PLoS One. 2026 Feb 10;21(2):e0340209. doi: 10.1371/journal.pone.0340209 (PMC12890140; doi:10.1371/journal.pone.0340209)
Supplement: S2 File — (DOCX) [file pone.0340209.s002.DOCX]

**Validation des échelles visuelles analogiques pour mesurer la satisfaction et le bien-être au travail**

**Acronym: SoWell-VAS**

**A propos de vous (à répondre une seule fois)**

**Age** : ………….

**Sexe** 0, Femme | 1, Homme

**Situation familiale** : □ Marié(e) / en couple □ Célibataire

**Nombre d’enfants** :

**Niveau d’études** :

□ brevet des collèges, collège ou inférieur

□ CAP/BEP

□ Baccalauréat

□ niveau BAC + 2 à BAC + 3

□ niveau BAC+ 5 : Master 2, Ingénieur, DEA ou DESS

□ niveau supérieur à BAC+ 5 : Doctorat ou autre

**Quel est votre statut professionnel actuel ?** 1, Cadre et profession intellectuelle supérieure | 2, Profession intermédiaire | 3, Agriculteur, exploitant | 4, Artisan, commerçant ou chef d'entreprise | 9 Professions de l'information, des arts et des spectacles | 5, ouvrier ou employé | 6, Elève ou étudiant | 7, à la recherche d'un emploi | 8, Retraité

**Quel est votre secteur d’activité ?** 1, Agriculture | 2, Industrie / construction |3, Tertiaire / Service / Autre (santé, commerce, administration, etc.)

*Si Tertiaire :* 1, Santé & social | 2, Enseignement & recherche |3, Administration | 4, Commerce et service | 5, Tourisme, hébergement, restauration | 6, Finance | 7, Transport | 8, Militaires / Forces de l’ordre / Pompiers | 9, Autres

**10. Avez-vous des fonctions d’encadrement ?** 1, Oui | 2, Non

**11. Nombre d’heures total de travail par semaine:** (tableau 0, 1 - 10, 10 - 20, 20 - 30, 30 - 40, 40 - 50, >50)

**12. Travail de nuit (actuel ou dernier emploi)**:

□ Non □ Fixe de nuit □ Travail posté □ Variable/aléatoire (Gardes ou astreintes ponctuelles)

□ Autres

**13. Nombre de weekend travaillés par mois ?** 0, 0 | 1, 1| 2, 2 | 3, 3 | 4, ≥4

**30. Nombre de cigarettes (tabac) par jour:** (0, 0 | 1, 1-4 | 2, 5-9 | 3, 10-14 | 4, 15-19 | 5, >20)

**31. Nombre de verres (alcool) par semaine:** (0, 0 | 1, 1-4 | 2, 5-9 | 3, 10-14 | 4, 15-19 | 5, >20)

**36. Taille (en cm)** (menu déroulant)

**37. Poids (en kg)** : (menu déroulant)

**33. Nombre d’heures assis par jour**: (menu déroulant entre 0 et 24, avec intervalles de 30min)

**34. Nombre d’heures d’activité physique de loisir par semaine** (menu déroulant entre 0 et 24, avec intervalles de 30min)

**Echelles visuelles analogiques - à renseigner deux fois à environ une semaine d’intervalle**

**Dans le cadre de votre travail**

**Quel est votre niveau de… ?** EVA 0 to 100, Min | Max

… stress

… soutien social

**A quelle fréquence êtes-vous confrontés à des conflits éthiques** (comportement qui entre en contradiction avec nos croyances) ? EVA 0 to 100, Jamais | Souvent

**D’une manière générale, quel est votre niveau de… ?** EVA 0 to 100 , Min | Max (OU mauvaise | excellente pour humeur / moral et qualité du sommeil)

… santé perçue

… stress à la maison

… fatigue

… qualité du sommeil

… humeur / moral

**Questionnaire validés - à renseigner deux fois à environ une semaine d’intervalle**

# Job Content questionnaire de Karasek

**Dans le cadre de votre travail, quel est votre niveau de… ?** EVA 0 to 100, Min | Max

… latitude décisionnelle (autonomie / contrôle dans votre travail)

… demande psychologique (charge de travail)

… soutien de votre chef (supérieur direct)

… soutien de votre hiérarchie (institution/entreprise)

… soutien de vos collègues

Entourer le score le plus approprié à chaque item :

| Pas du tout d’accord | Pas d’accord | D’accord | Tout à fait d’accord | |
| --- | --- | --- | --- | --- |
| **1** | **2** | **3** | **4** | |
| Q1 - Dans mon travail, je dois apprendre des choses nouvelles | | | | **1 2 3 4** |
| Q2 - Dans mon travail, j’effectue des tâches répétitives | | | | **1 2 3 4** |
| Q3 - Mon travail me demande d’être créatif | | | | **1 2 3 4** |
| Q4 - Mon travail me permet de prendre souvent des décisions moi-même | | | | **1 2 3 4** |
| Q5 - Mon travail demande un haut niveau de compétence | | | | **1 2 3 4** |
| Q6 - Dans ma tâche, j’ai très peu de libertés pour décider comment je fais mon travail | | | | **1 2 3 4** |
| Q7 - Dans mon travail, j’ai des activités variées | | | | **1 2 3 4** |
| Q8 - J’ai la possibilité d’influencer le déroulement de mon travail | | | | **1 2 3 4** |
| Q9 - J’ai l’occasion de développer mes compétences professionnelles | | | | **1 2 3 4** |
| Q10 - Mon travail me demande de travailler très vite | | | | **1 2 3 4** |
| Q11 - Mon travail me demande de travailler intensément | | | | **1 2 3 4** |
| Q12 - On me demande d’effectuer une quantité de travail excessive | | | | **1 2 3 4** |
| Q13 - Je dispose du temps pour exécuter correctement mon travail | | | | **1 2 3 4** |
| Q14 - Je reçois des ordres contradictoires de la part d’autres personnes | | | | **1 2 3 4** |
| Q15 - Mon travail demande de longues périodes de concentration intense | | | | **1 2 3 4** |
| Q16 - Mes tâches sont souvent interrompues avant d’être achevées, nécessitant de les reprendre plus tard | | | | **1 2 3 4** |
| Q17 - Mon travail est très bousculé | | | | **1 2 3 4** |
| Q18 - Attendre le travail de collègues ou d’autres départements ralentit souvent mon propre travail | | | | **1 2 3 4** |
| Q19 - Mon supérieur se sent concerné par le bien-être de ses subordonnés | | | | **1 2 3 4** |
| Q20 - Mon supérieur prête attention à ce que je dis | | | | **1 2 3 4** |
| Q21 - Mon supérieur m’aide à mener ma tâche à bien | | | | **1 2 3 4** |
| Q22 - Mon supérieur réussit facilement à faire collaborer ses subordonnés | | | | **1 2 3 4** |
| Q23 - Les collègues avec qui je travaille sont des gens professionnellement compétents | | | | **1 2 3 4** |
| Q24 - Les collègues avec qui je travaille me manifestent de l’intérêt | | | | **1 2 3 4** |
| Q25 - Les collègues avec qui je travaille sont amicaux | | | | **1 2 3 4** |
| Q26 - Les collègues avec qui je travaille m’aident à mener les tâches à bien | | | | **1 2 3 4** |
|  | | | | |

# Hospital Anxiety and Depression scale (HAD)

**D’une manière générale, quel est votre niveau de… ?** EVA 0 to 100, Min | Max ou Mauvaise | Excellente pour humeur

… humeur

… anxiété

… dépression

Entourer le score le plus approprié à chaque item :

| **A** | **Je me sens tendu, énervé :**  - la plupart du temps  - souvent  - de temps en temps  - jamais | **3 2 1 0** |  | **H** | **J'ai une sensation de peur, comme si quelque chose d'horrible allait m'arriver :** - oui nettement  - oui mais ce n'est pas trop grave  - un peu, mais cela ne m'importe pas  - pas du tout | **3 2 1 0** |
| --- | --- | --- | --- | --- | --- | --- |
| **B** | **J'ai toujours autant de plaisir à faire les choses qui me plaisent :**  - oui toujours  - le plus souvent  - de plus en plus rarement  - tout est difficile | **0 1 2 3** |  | **I** | **Je sais rire et voir le bon côté des choses :**  - toujours autant  - plutôt moins  - nettement moins  - pas du tout | **0 1 2 3** |
| **C** | **Je me sens ralenti :**  - pratiquement tout le temps  - Très souvent  - quelquefois  - pas du tout | **3 2 1 0** |  | **J** | **Je me fais souvent du souci :**  - très souvent  - assez souvent  - occasionnellement  - très occasionnellement | **3**  **2 1 0** |
| **D** | **J'éprouve des sensations d'angoisse et j'ai une boule dans la gorge ou l'estomac noué :**  - très souvent  - assez souvent  - parfois  - jamais | **3 2 1 0** |  | **K** | **Je me sens heureux :**  - jamais  - pas souvent  - quelquefois  - la plupart du temps | **3 2 1 0** |
| **E** | **J'ai perdu l'intérêt pour mon apparence :**  - totalement  - je n'y fais pas attention  - je n'y fais plus assez attention  - j'y fais attention comme d' habitude | **3 2 1 0** |  | **L** | **Je peux rester tranquillement assis à ne rien faire et me sentir détendu :**  - jamais  - rarement  - oui en général  - oui, quoi qu'il arrive | **0 1 2 3** |
| **F** | **J'ai la bougeotte et n'arrive pas à tenir en place :**  - oui, c'est tout à fait le cas  - plutôt moins qu'avant  - beaucoup moins qu'avant  - pas du tout | **3 2 1 0** |  | **M** | **Je m'intéresse à la lecture d'un bon livre ou à un bon programme radio ou télé :**  - souvent  - assez souvent  - rarement  - pratiquement jamais | **0 1 2 3** |
| **G** | **J'envisage l'avenir avec optimisme :**  - comme d'habitude  - plutôt moins qu'avant  - beaucoup moins qu'avant  - pas du tout | **0 1 2 3** |  | **N** | **J'éprouve des sensations soudaines de panique :**  - très souvent  - assez souvent  - rarement  - jamais | **3 2 1 0** |
